# Role of the nonhelical tailpiece of myosin-II in regulating filament architecture and function

**DOI:** 10.1083/jcb.202501234

**Published:** 2026-06-25

**Authors:** Kangji Wang, Shi Shu, Xiong Liu, Erfei Bi

**Affiliations:** 1 Department of Cell and Developmental Biology, Perelman School of Medicine, University of Pennsylvania, Philadelphia, PA, USA; 2 Laboratory of Cell Biology, National Heart, Lung, and Blood Institute, National Institutes of Health, Bethesda, MD, USA

## Abstract

Mammalian nonmuscle myosin-II isoforms (NM-IIA, NM-IIB, and NM-IIC) each contain a nonhelical tailpiece (NHT) at their C terminus. Stop-codon mutations in the NHT of NM-IIA are linked to diseases such as macrothrombocytopenia. However, the role of the NHT in NM-II filament assembly and function remains poorly understood. Here, we show that NM-II isoforms lacking the NHT, including disease-associated NM-IIA truncations, form enlarged bipolar filaments with reduced bare zones. NHT length emerges as a key determinant of filament size. Moreover, NM-IIA NHT truncations generate stress fibers composed of enlarged bipolar filaments that exhibit reduced FRAP recovery and an increased tendency to aggregate, resulting in impaired cell migration. We further provide in vivo evidence that NM-IIA assembles into bipolar filaments in the absence of RLC phosphorylation, a property enhanced by NHT deletion. Together, these findings establish the NHT as a critical regulator of NM-II filament architecture, dynamics, and function and provide mechanistic insight into NM-IIA NHT-associated diseases.

## Teaser

Wang et al. show that deletion of the nonhelical tailpiece (NHT) in myosin-II results in enlarged bipolar filaments with reduced bare zones, decreased disassembly, and impaired cell migration, providing mechanistic insight into disease-associated stop-codon mutations in the NHT of myosin-IIA.

## Introduction

Nonmuscle myosin-IIs (NM-IIs) play essential roles in cytokinesis, cell migration, and cell adhesion during tissue morphogenesis (Dulyaninova and Bresnick, 2013; Pecci et al., 2018; Shutova and Svitkina, 2018; Vicente-Manzanares et al., 2009; Wang et al., 2020). These functions depend on the dynamic assembly and disassembly of NM-II filaments. Mammalian cells contain three NM-II isoforms (NM-IIA, NM-IIB, and NM-IIC), each consisting of two heavy chains (HCs), two essential light chains (ELCs), and two regulatory light chains (RLCs). Each HC has an N-terminal globular head (motor domain) with actin binding and ATPase activity, a short neck region containing two IQ motifs that bind ELC and RLC, a helical tail that mediates HC–HC interactions to form the NM-II backbone, and a short nonhelical tailpiece (NHT) of 33, 43, and 47 amino acids (aa) in NM-IIA, NM-IIB, and NM-IIC, respectively. Understanding how monomers assemble into bipolar filaments of defined size (Billington et al., 2013; Liu et al., 2017) and how these filaments disassemble back into monomers has been a central focus of NM-II research.

The role of RLC phosphorylation in promoting myosin-II filament assembly has been extensively studied in vitro. Similar to smooth muscle myosin-II (Burgess et al., 2007; Suzuki et al., 1982; Trybus et al., 1982), NM-II monomers exist in either an unfolded (extended, 6S) or a folded (10S) conformation (Craig et al., 1983; Kendrick-Jones et al., 1987). In the 10S form, the two HC heads associate, and the helical tail folds back to interact with the RLC and other regions, thereby inhibiting actin binding and ATPase activity (Heissler et al., 2021; Kiboku et al., 2013; Olney et al., 1996; Salzameda et al., 2006; Wendt et al., 1999; Wendt et al., 2001; Yang et al., 2019). RLC phosphorylation induces tail unfolding, thereby converting the monomer from the 10S to the 6S conformation, activating ATPase activity, and promoting filament assembly (Craig et al., 1983; Kendrick-Jones et al., 1987). Recent studies indicate that both RLC-phosphorylated and RLC-nonphosphorylated NM-IIs can exist not only as folded monomers but also as folded antiparallel dimers and tetramers, in the presence or absence of ATP (1 mM ATP-depolymerized filaments formed by RLC-unphosphorylated myosin in vitro). RLC phosphorylation is thought to promote filament assembly in vitro by enhancing the unfolding of folded antiparallel dimers and tetramers in addition to folded monomers (Liu et al., 2017). Further analyses suggest that folded antiparallel tetramers may serve as principal building blocks of NM-II filaments in vitro (Liu et al., 2018). In this proposed model, RLC phosphorylation promotes unfolding of these tetramers to initiate bipolar filament assembly with a bare zone, after which additional folded tetramers associate with and unfold at the bare zone to generate mature bipolar filaments (Liu et al., 2018). In contrast, the role of RLC phosphorylation in NM-II filament assembly in vivo remains unclear.

The role of the NHT in NM-II filament assembly is even less well defined, both in vitro and in vivo. Stop-codon mutations in the NHT (residues 1,933, 1,941, and 1,945) of NM-IIA cause macrothrombocytopenia, characterized by fewer and larger platelets, and, in some cases, deafness, cataracts, and nephritis (Asensio-Juarez et al., 2020; Heath et al., 2001; Pecci et al., 2018; Seri et al., 2003), underscoring the physiological importance of this region. Previous studies using recombinant C-terminal tail fragments (rods) have yielded conflicting results: deletion or truncation of the NHT inhibits rod assembly in some contexts (Franke et al., 2005; Hodge et al., 1992) but enhances assembly in others (Ronen and Ravid, 2009). These discrepancies may reflect differences among NM-II isoforms or variations in tail rod length and composition. Consistent with this idea, swapping NHTs between NM-II isoforms alters paracrystal structure (Ronen and Ravid, 2009). However, because tail rods assemble into paracrystals rather than bipolar filaments or folded intermediates, it remains unclear how these observations relate to the physiological role of the NHT.

In vivo analyses of NHT deletions or chimeras have largely focused on localization and solubility. Deletion of the NHT from NM-IIA does not affect its localization to the leading edge in mouse embryonic fibroblasts (Ronen and Ravid, 2009) or migrating HeLa cells (Breckenridge et al., 2009). In contrast, chimeric NM-IIA– or NM-IIB–containing NHTs from different isoforms adopt localization patterns resembling that of the NHT donor (Ronen and Ravid, 2009). Although NHT-deleted NM-IIA is more insoluble, suggesting overassembly (Breckenridge et al., 2009), the architecture and function of filaments formed by NHT-less NM-IIA have not been examined.

To address these gaps, we purified full-length NM-II isoforms, NHT chimeras, and NM-IIA variants with distinct NHT lengths or aa compositions from Sf9 cells. We show that the NHT length is a major determinant of NM-II filament size. NHT truncations, including those associated with *MYH9*-related disease (*MYH9*-RD), promote the formation of enlarged bipolar filaments with reduced bare zones in vitro. In human osteosarcoma-derived U2OS cells, NM-IIA NHT variants lacking the NHT or carrying disease-associated truncations likewise form enlarged bipolar filaments with reduced FRAP recovery and an increased tendency to aggregate, particularly during filament disassembly. Importantly, NHT-less NM-IIA impairs cell migration. Together, these findings identify the NHT as a critical regulator of NM-II filament assembly and function and provide a mechanistic framework for understanding disease phenotypes caused by NHT truncations in *MYH9*-RD.

## Results

### Essential role of the NHT in preventing uncontrolled assembly of NM-II into large filaments

**Figure 1.**
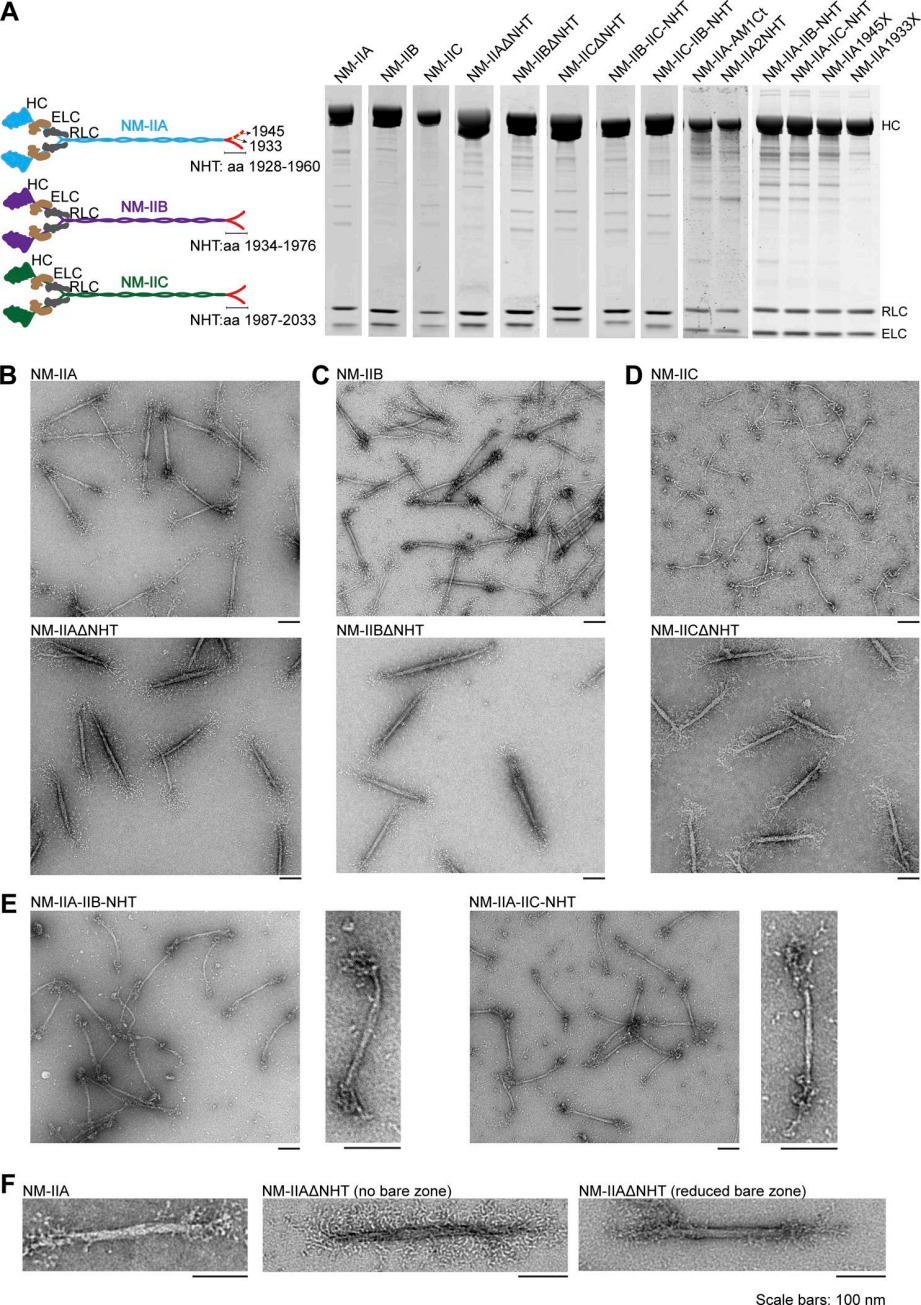
**Essential role of the NHT in preventing uncontrolled NM-II assembly into large filaments. (A)** Electrophoretic analysis of purified recombinant NM-IIs. The first 1–8 lanes show samples run on 10% SDS-PAGE gels, while the 9–14 lanes show samples run on 4–12% gradient gels. All gels were stained with Coomassie blue. **(B–E)** Electron micrographs of polymerized NM-IIA and NM-IIAΔNHT (B), NM-IIB and NM-IIBΔNHT (C), NM-IIC and NM-IICΔNHT (D), and NM-IIA-IIB-NHT and NM-IIA-IIC-NHT (E). **(F)** Individual filaments of NM-IIA and NM-IIAΔNHT in higher magnification. Myosins were polymerized overnight on ice in a buffer containing 10 mM MOPS (pH 7.0), 2 mM MgCl_2_, 150 mM NaCl, and 0.1 mM EGTA. Note: The image shown in Fig. 1 C (top panel) is identical to that shown in Fig. 3 A (left panel), and the image shown in Fig. 1 D (top panel) is identical to that shown in Fig. 3 C (left panel). These images are included in multiple figures to facilitate comparison of the relevant data. Source data are available for this figure: SourceData F1.

To investigate the biochemical role of the NHT in NM-II filament assembly and understand the pathogenetic mechanisms of stop-codon mutations in the NHT of NM-IIA, we co-expressed various N-terminal FLAG-tagged HCs with the RLC and ELC in Sf9 cells, and purified the recombinant NM-IIs using anti-FLAG affinity chromatography (Fig. 1 A) (Billington et al., 2013; Liu et al., 2017; Liu et al., 2018).

**Figure 2.**
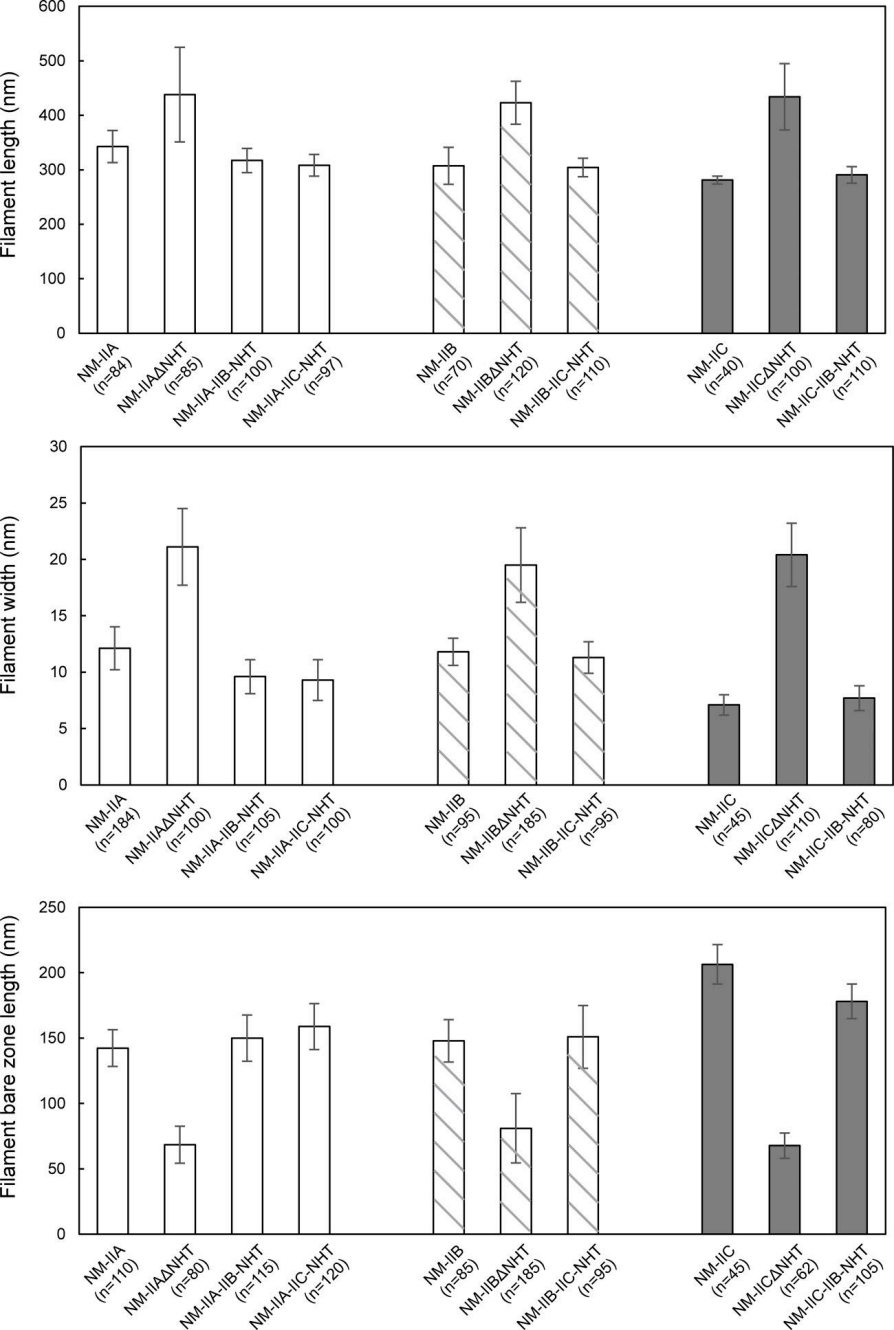
**Quantitative analyses of filament dimensions of NM-IIA, NM-IIB, and NM-IIC and their NHT variants.** Comparison of filament length, width, and bare-zone dimensions of WT NM-IIA, NM-IIB, and NM-IIC, alongside NHT chimeras and NHT deletion variants. Filament lengths, widths, and bare-zone lengths were quantified from electron micrographs similar to those in Figs. 1 and 3. Data are presented as the mean ± SD.

We examined by negative-staining electron microscopy (EM) the structures of filaments assembled overnight at 4°C in the absence of ATP by RLC-unphosphorylated NM-IIs with or without their NHTs. As expected, different isoforms formed their characteristic bipolar filaments (Fig. 1, B–D and Fig. 2) (Billington et al., 2013; Liu et al., 2017; Liu et al., 2018). Strikingly, deletion of the NHT produced marked alterations in filament architecture across all isoforms, including a shortened bare zone and increased filament length and width (Fig. 1, B–D and Fig. 2; note that only filaments with a clearly identifiable bare zone were included in the quantitative analyses). Detectable bare zones were present in only 15%, 55%, and 13% of filaments formed by NM-IIAΔNHT, NM-IIBΔNHT, and NM-IICΔNHT, respectively; the remaining filaments lacked discernible bare zones, likely due to filament staggering.

**Figure 3.**
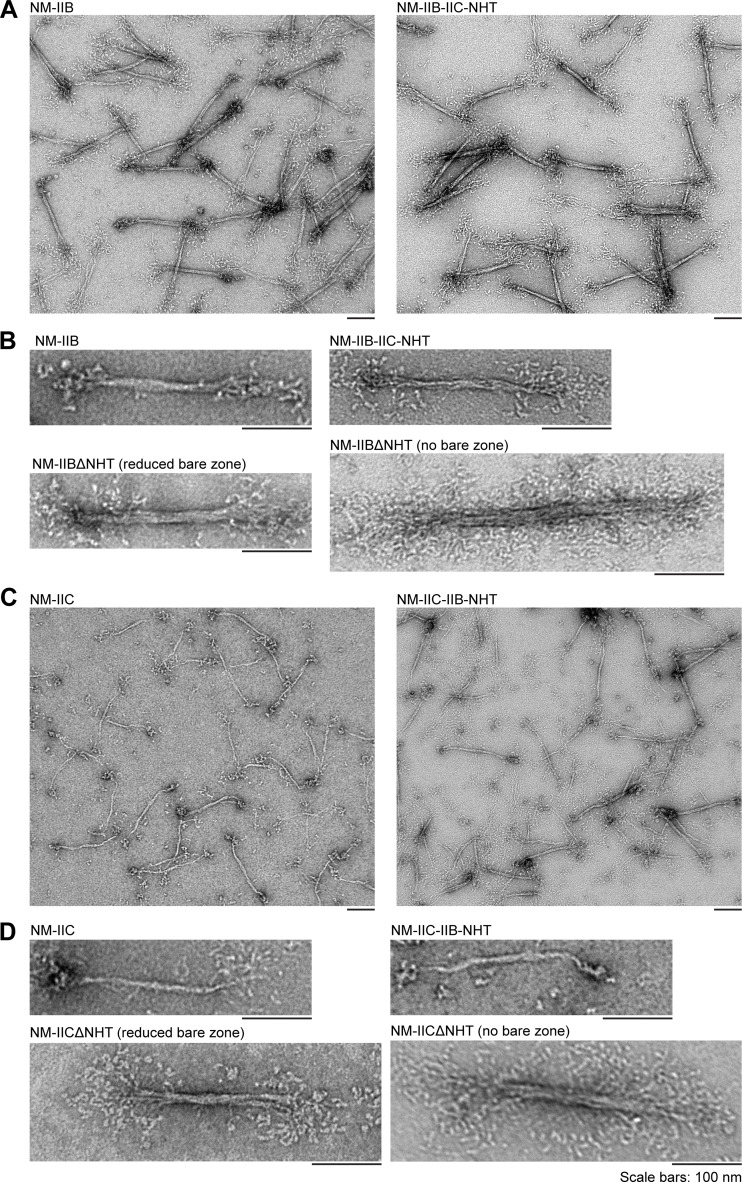
**NHTs of NM-IIB and NM-IIC control their filament size. (A)** Electron micrographs of polymerized NM-IIB and NM-IIB-IIC-NHT. **(B)** Zoomed-in view of polymerized NM-IIB, NM-IIB-IIC-NHT, and NM-IIBΔNHT. **(C)** Electron micrographs of polymerized NM-IIC and NM-IIC-IIB-NHT. **(D)** Zoomed-in view of polymerized NM-IIC, NM-IIC-IIB-NHT, and NM-IICΔNHT. Myosins were polymerized under the same conditions as described in Fig. 1. Note: The image shown in Fig. 3 A (left panel) is identical to that shown in Fig. 1 C (top panel), and the image shown in Fig. 3 C (left panel) is identical to that shown in Fig. 1 D (top panel). These images are included in multiple figures to facilitate comparison of the relevant data.

To determine whether the NHT sequence influences filament morphology (length, width, and bare-zone length), we expressed and purified NM-II chimeras with swapped NHTs (NM-IIA-IIB-NHT, NM-IIA-IIC-NHT, NM-IIB-IIC-NHT, and NM-IIC-IIB-NHT) (Fig. 1 A). The filament morphology characteristic of NM-IIB and NM-IIC was largely retained in the NM-IIB-IIC-NHT and NM-IIC-IIB-NHT chimeras (Figs. 2 and 3). In contrast, a moderate change in filament dimensions was observed for the NM-IIA isoform in the NM-IIA-IIB-NHT and NM-IIA-IIC-NHT chimeras (Fig. 1, E and F; and Fig. 2). This difference is likely due to the larger length discrepancy between the NM-IIA NHT (33 aa) and those of NM-IIB (43 aa) or NM-IIC (47 aa) (see next section for further analysis). Notably, our results with full-length NM-IIs differ from previous findings using NM-II tail fragments, which suggested that the NHT sequence dictates isoform-specific morphology of the paracrystals (Ronen and Ravid, 2009).

Collectively, these data demonstrate that the NHT is essential for preventing uncontrolled NM-II assembly, with its presence or absence being more important than its specific sequence.

### NHT length as a major determinant of NM-IIA filament size

**Figure 4.**
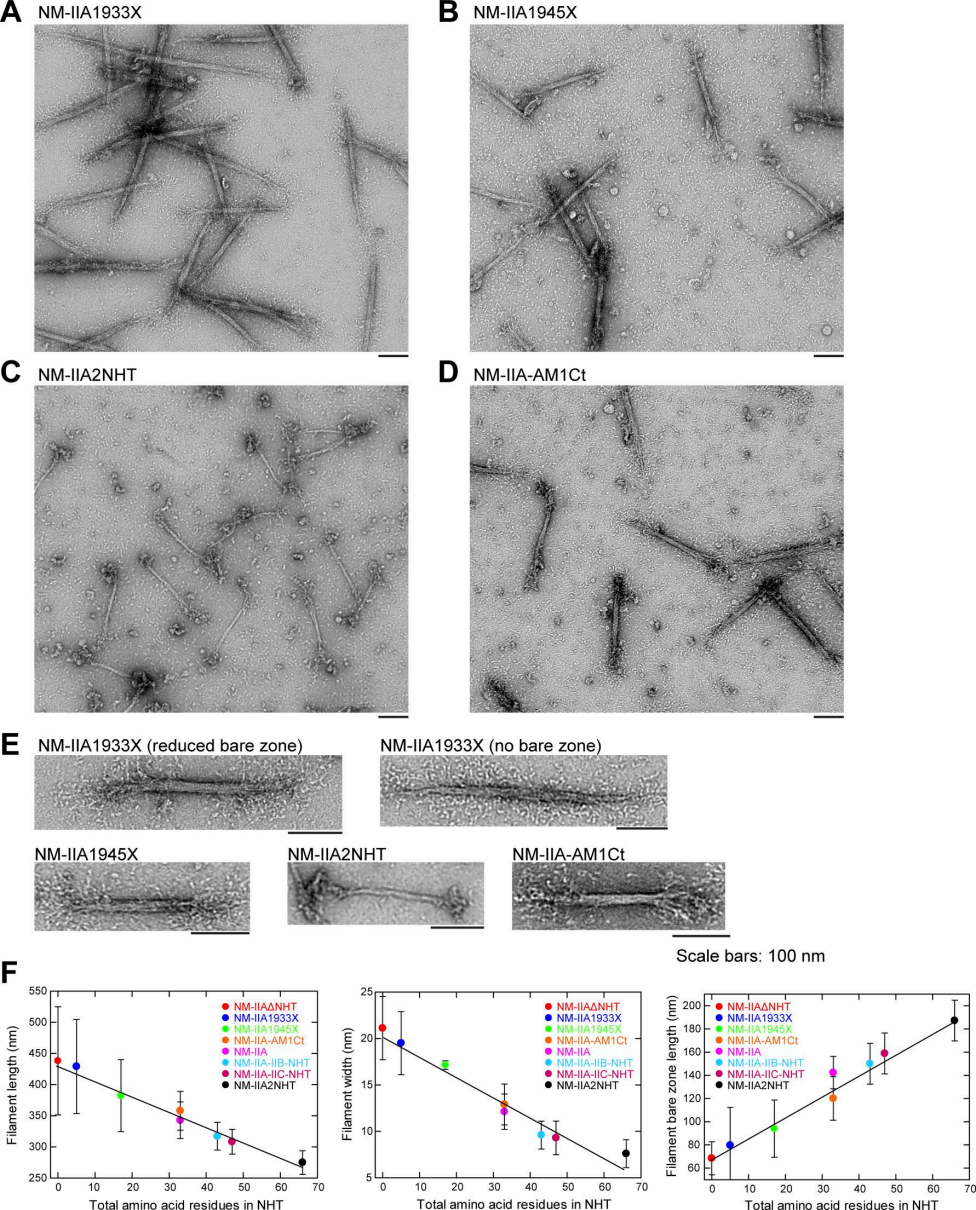
**NHT length as a major determinant of NM-IIA filament size. (A–D)** Electron micrographs of polymerized NM-IIA1933X (A), NM-IIA1945X (B), NM-IIA2NHT (C), and NM-IIA-AM1Ct (D). **(E)** Individual filaments of these NM-IIA NHT variants in higher magnification. Myosins were polymerized under the same conditions as in Fig. 1. **(F)** Filament dimensions of WT NM-IIA and its NHT variants plotted against the total number of aa in their NHTs. Filament dimensions were measured from electron micrographs (Figs. 1 and 4) using MetaMorph. Data are presented as the mean ± SD.

The analysis of NM-IIA chimeras raised the possibility that NHT length may regulate filament size. To explore this, we constructed and purified several NM-IIA variants with different NHT lengths (Fig. 1 A). In addition to the wild-type (WT) and NM-IIAΔNHT (deleted after residue P1927), we constructed two NM-IIA variants with NHTs truncated after residues R1933 and E1945 (NM-IIA1933x, NM-IIA1945x). These truncations were based on genetic mutations in *MYH9* that cause macrothrombocytopenia (Asensio-Juarez et al., 2020; Heath et al., 2001; Kelley et al., 2000; Pecci et al., 2018; Seri et al., 2000; Seri et al., 2003). We also constructed an NM-IIA variant with its NHT duplicated in tandem (NM-IIA2NHT). EM and quantitative analyses showed that NM-IIA1945x and NM-IIA1933x, like NM-IIAΔNHT, formed larger, thicker filaments with reduced or no detectable bare zones (Fig. 4, A, B, E, and F). In contrast, NM-IIA2NHT formed smaller, thinner filaments with more prominent bare zones (Fig. 4, C, E, and F). A similar, though more moderate, effect was observed in the NM-IIA-IIB-NHT and NM-IIA-IIC-NHT chimeras, where the shorter IIA NHT was replaced with the longer IIB and IIC NHTs, respectively (Fig. 4 F).

To further examine the impact of NHT sequence, we constructed an NM-IIA variant in which the 33-aa NM-IIA NHT (FVVPRRMARKGAGDGSDEEVDGKADGAEAKPAE) was replaced with a 33-aa nonhelical tail fragment (RGRGGPGPAPPGGMARGGMMPPRGRAGPPPPGM) from *Acanthamoeba* myosin 1C (NM-IIA-AM1Ct). Strikingly, NM-IIA-AM1Ct formed filaments with dimensions similar, but not identical, to NM-IIA (Fig. 4, D–F). The bipolar filaments formed by NM-IIA-AM1Ct were comparable or slightly larger in length and width but had a reduced bare zone (Fig. 4 F).

Together, these data suggest that NHT length is a major determinant of NM-IIA filament size, with a minor contribution from its specific sequence.

### NHT is dispensable for the initial assembly of NM-IIA filaments

**Figure 5.**
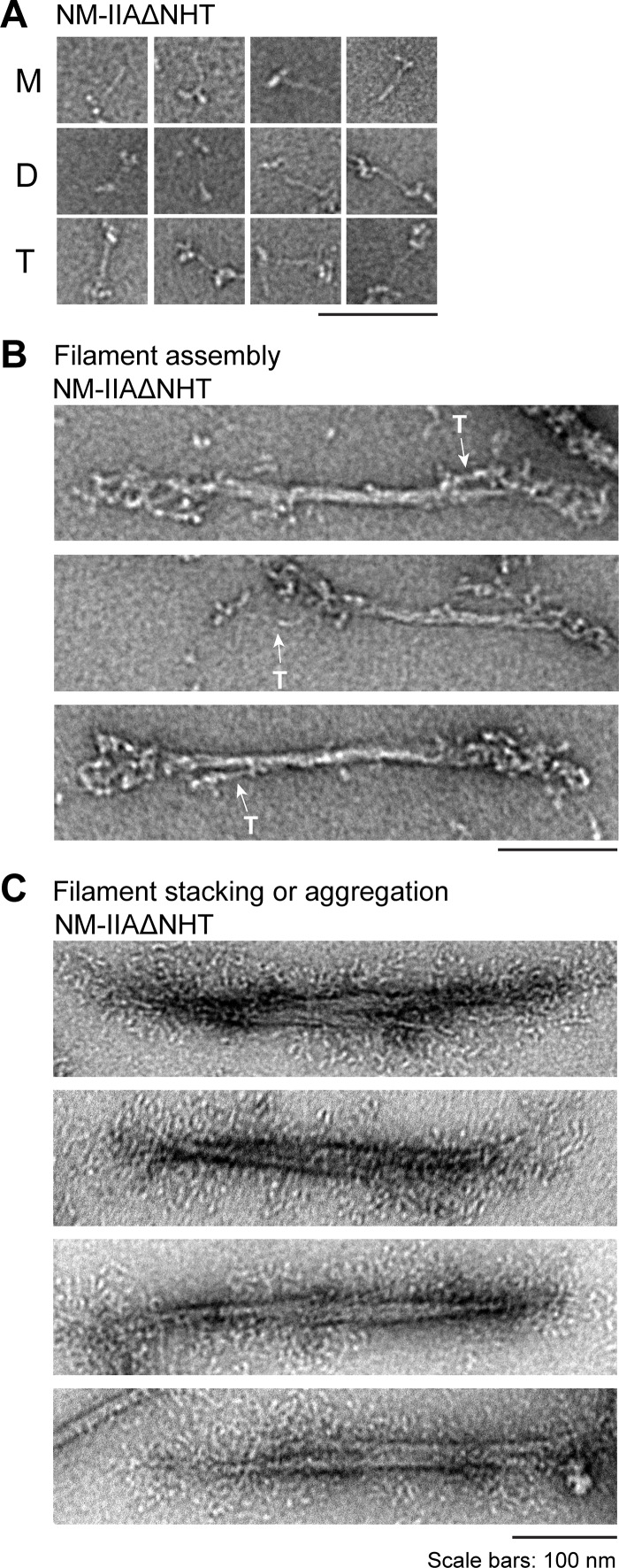
**Intermediate structures formed during NM-IIAΔNHT polymerization. (A)** Representative images of folded monomers (M), antiparallel dimers (D), and tetramers (T). **(B)** Arrows indicate antiparallel tetramers of different lengths along growing filaments. Myosin was polymerized for 4 s and immediately fixed with glutaraldehyde to visualize polymerization intermediates (A and B). **(C)** Stacked filaments lacking visible bare zones. Samples were polymerized overnight.

To understand how NM-IIAΔNHT assembles into larger filaments, we examined its polymerization by rapidly diluting RLC-unphosphorylated NM-IIAΔNHT from high-salt (600 mM NaCl) buffer into low-salt (150 mM NaCl) polymerization buffer in the absence of ATP. The reaction was allowed to proceed for only 4 s at room temperature, after which it was fixed to trap assembly intermediates and visualized by negative-staining EM (Liu et al., 2018). We found that NM-IIAΔNHT polymerized into bipolar filaments in a manner similar to WT NM-IIA (Liu et al., 2018). In brief, NM-IIAΔNHT formed folded monomers, as well as folded antiparallel dimers and tetramers (Fig. 5 A). In addition, folded or partially unfolded antiparallel tetramers were associated with the bare zones of bipolar filaments of various sizes (Fig. 5 B, arrows). These tetramers were thought to unfold and incorporate into mature bipolar filaments, as previously suggested (Liu et al., 2018).

For WT NM-IIA, mature filaments formed after overnight polymerization exhibited typical dimensions, with an average bare-zone length of ∼142 nm (Fig. 4 F). In contrast, NM-IIAΔNHT formed larger bipolar filaments with a shorter average bare-zone length of ∼69 nm (Fig. 4 F), which is approximately the length of a folded antiparallel tetramer (∼65 nm) (Fig. 5, A and B) (Liu et al., 2018). We also observed filament stacking or aggregation composed of two or more of these larger bipolar filaments with variable staggering, resulting in long filaments lacking bare zones (Fig. 5 C). Together, these data suggest that while the NHT is not required for the initial assembly of NM-IIA bipolar filaments, it is essential for preventing excessive assembly at the bare zone and filament aggregation, thereby ensuring proper filament structure.

### Deletion of the NHT does not affect the critical concentration for NM-II polymerization in the absence of ATP

**Figure 6.**
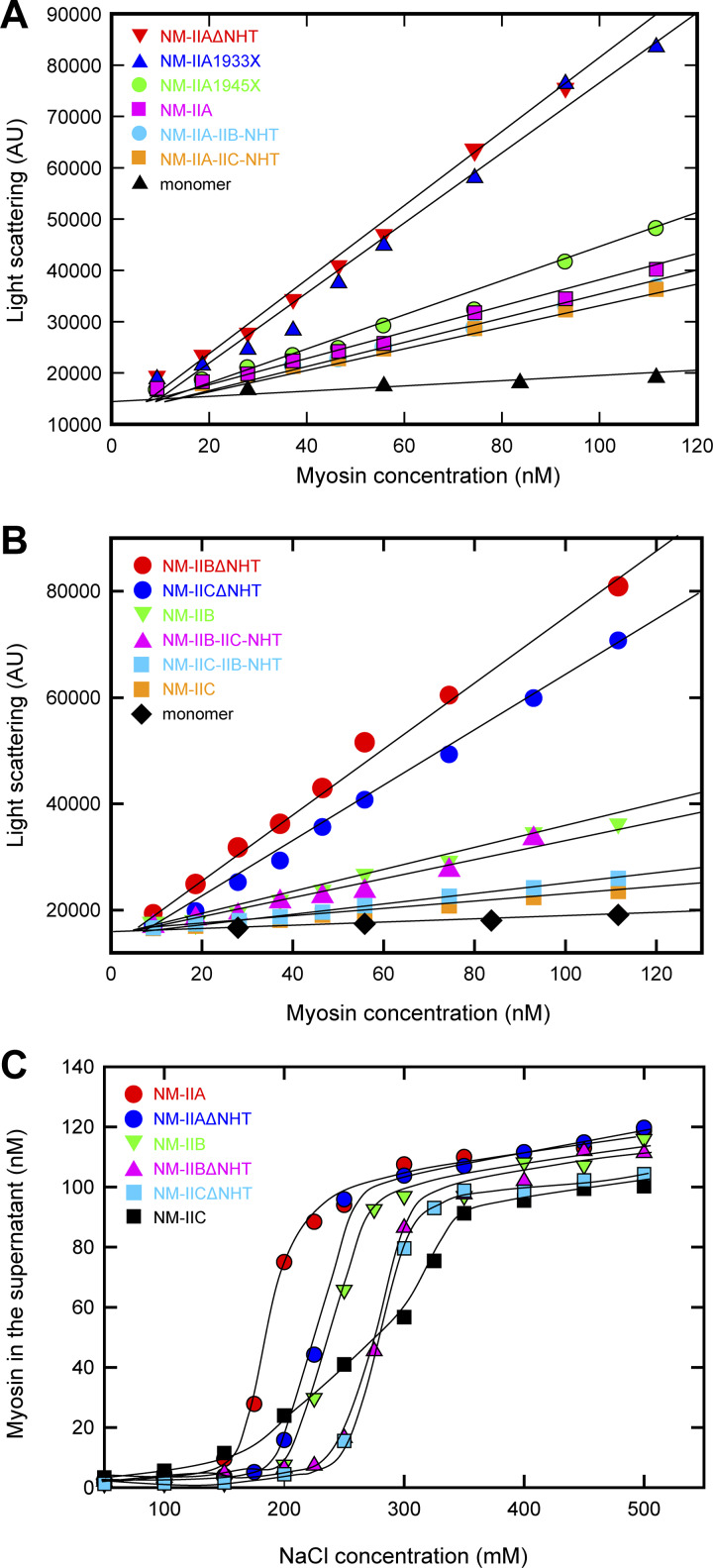
**NHT deletion does not affect the CC for NM-II polymerization in the absence of ATP. (A and B)** CCs of NM-IIs for polymerization. NM-IIs at varying concentrations were incubated overnight as described in Fig. 1, and warmed to room temperature for 30 min, and light scattering was measured at 365 nm using a PTI fluorimeter. CC was determined from the intersection of the plotted curves for polymerized and monomeric myosin. Data represent the average of two independent experiments. **(C)** Effect of NHT deletion on NM-II solubility. Full-length and NHT-deleted NM-IIs (150 nM) were incubated as in Fig. 1 with varying NaCl concentrations. After centrifugation at 400,000 × *g* for 20 min to separate polymerized (pellet) and soluble (supernatant) myosin, the amounts of polymerized and unpolymerized myosin were determined by Coomassie-stained SDS-PAGE. Data represent the average of two independent experiments.

To further investigate the mechanism of NM-IIAΔNHT polymerization, we monitored its assembly as a function of myosin concentration in the absence of ATP by light scattering. For both WT and all NHT variants of NM-IIA, light scattering increased linearly with myosin concentration, though the rate of increase was higher for the NHT truncation variants (Fig. 6 A). This is consistent with NHT truncation variants assembling into larger bipolar filaments or staggered filament aggregates (Figs. 1, 4, and 5). The intercepts of their light-scattering plots with the monomeric myosin light-scattering plot define the minimal myosin concentration required for polymerization, i.e., the critical concentration (CC). Remarkably, the CCs for the WT, NHT-deleted, NHT-chimeric, and NHT-truncated NM-IIA variants were nearly identical, around 10 nM. A similar effect of the NHT deletion on the CC of light scattering was observed for NM-IIB and NM-IIC (Fig. 6 B). Thus, deletion of the NHT does not affect the CC of NM-II polymerization in the absence of ATP.

We also monitored polymerization of WT and the NHT-deleted variants of NM-IIs in various NaCl concentrations (50–500 mM) by measuring the myosin concentration in the supernatant after pelleting the filamentous myosin at 400,000 g for 20 min. Deletion of the NHT increased the NaCl concentrations required for 50% depolymerization from 180, 240, and 270 mM to 235, 275, and 280 mM for NM-IIA, NM-IIB, and NM-IIC, respectively (Fig. 6 C). These results suggest that the NHT-less NM-IIs are more likely to form stable filament structures, presumably due to the absence of NHT-mediated steric hindrance in the bare-zone region of bipolar filaments, compared with the WT NM-IIs at a given NaCl concentration. This suggests that the NHT likely weakens the association between building blocks, such as the unfolded tetramers in the filaments, through steric hindrance.

In summary, deletion of the NHT does not change the CC in the absence of ATP. This finding contrasts with a previous study in which NHT deletion in chicken NM-II caused a ∼50-fold increase in CC and reduced filament assembly (Hodge et al., 1992). This discrepancy is likely due to the fact that our study used full-length NM-IIs, whereas the earlier study utilized an NM-II rod fragment (Hodge et al., 1992).

### NHT truncations in NM-IIA increase bipolar filament size and reduce FRAP recovery in vivo

**Figure 7.**
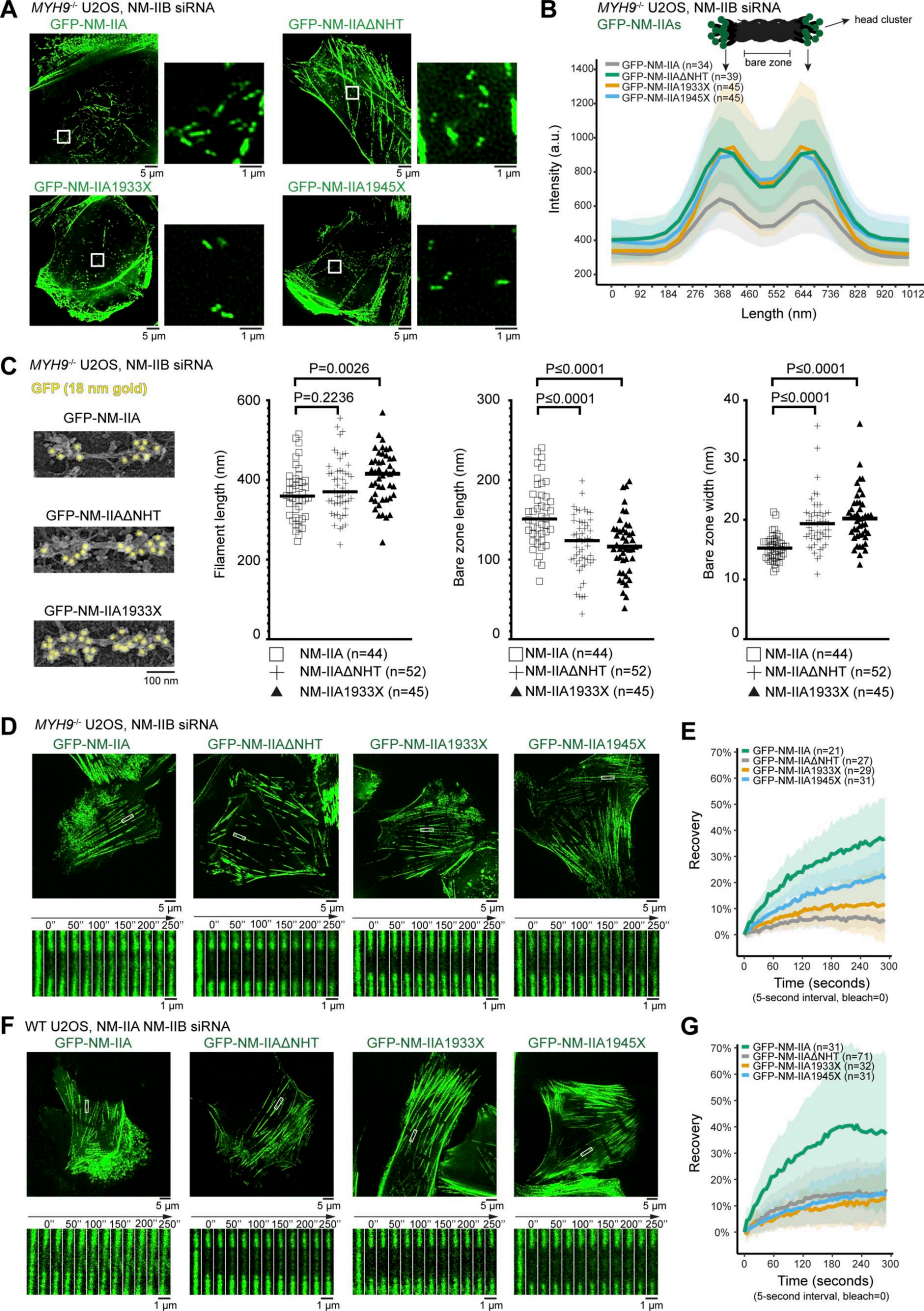
**NHT truncations in NM-IIA lead to the formation of enlarged bipolar filaments in vivo. (A)** iSIM images of NM-IIB–knockdown, NM-IIA-KO U2OS cells expressing GFP-NM-IIA, GFP-NM-IIAΔNHT, GFP-NM-IIA1933X, or GFP-NM-IIA1945X. Individual bipolar filaments in the cytosol are highlighted (white boxes), with enlarged views shown in adjacent panels. **(B)** Line-scan quantification of GFP intensity along myosin heads and bare zones from images as in A. Only GFP clusters lacking NM-IIB were included. Data are presented as the mean ± SD. **(C)** Immunogold-labeling PREM analysis of bipolar filaments from NM-IIB–knockdown, NM-IIA-KO U2OS cells expressing GFP-NM-IIA, GFP-NM-IIAΔNHT, or GFP-NM-IIA1933X. GFP was labeled with 18-nm gold particles (yellow). Filament length, bare-zone length, and bare-zone width were measured from >40 individual filaments per condition and plotted. *n* indicates the number of myosin filaments analyzed for each of the NM-IIA NHT variant. P values were determined using a two-sided Mann–Whitney U test. **(D)** FRAP analysis of GFP-NM-IIA, GFP-NM-IIAΔNHT, GFP-NM-IIA1933X, and GFP-NM-IIA1945X in NM-IIB–knockdown, NM-IIA-KO U2OS cells. 72 h after NM-IIB siRNA treatment, a region of the ventral stress fiber was photobleached, and fluorescence recovery was followed over time. Enlarged views of boxed regions are shown below. Images were acquired at 5-s intervals. **(E)** Fluorescence recovery was quantified as the percentage of prebleach intensity. **(F and G)** Corresponding FRAP experiments were performed in WT U2OS cells with NM-IIA and NM-IIB knocked down. Recovery was quantified as in E. Data are presented as the mean ± SD.

**Figure S1.**
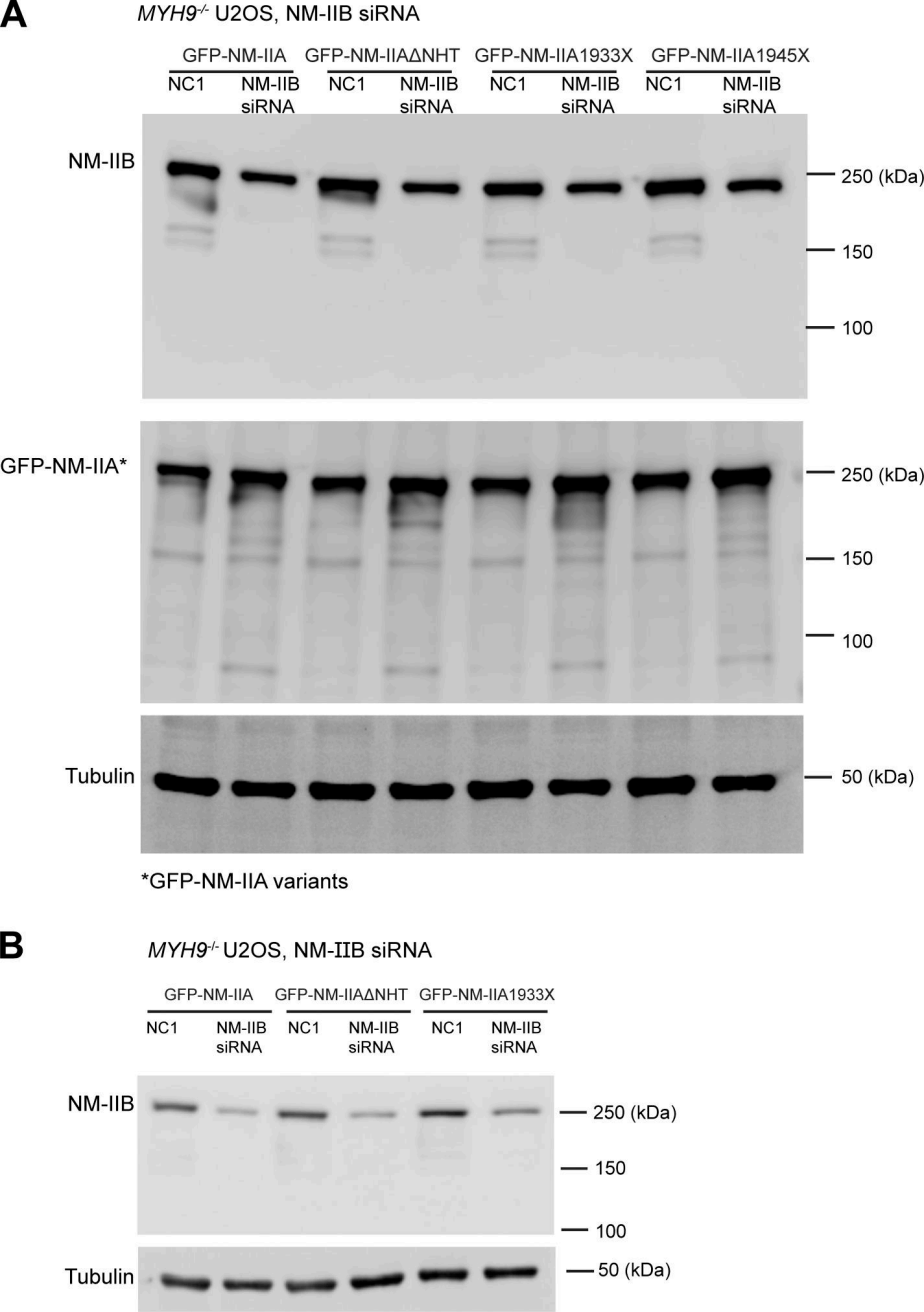
**Western blot analyses of NM-IIB knockdown efficiency and GFP-tagged NM-IIA NHT variant expression levels in NM-IIA-KO U2OS cells. Related to Figs. 7 and 8. (A)** Knockdown efficiency of NM-IIB and expression levels of GFP-NM-IIA NHT variants (indicated by GFP-NM-IIA*) in NM-IIA-KO U2OS cells used for iSIM analyses (related to Fig. 7, A and B). **(B)** Knockdown efficiency of NM-IIB in NM-IIA-KO U2OS cells used for immunogold-labeling PREM to assess filament size and for immunostaining of pRLC and ppRLC (related to Fig. 7 C and Fig. 8 D). Source data are available for this figure: SourceData FS1.

To examine the effect of NHT truncations on NM-II assembly and dynamics in vivo, we expressed various N-terminally GFP-tagged NM-IIA NHT truncation variants from the CMV promoter in *MYH9*-knockout (*MYH9*^*−/−*^, NM-IIA-KO) U2OS cells (Fig. 7 A). Endogenous NM-IIB was also knocked down to minimize its influence on NM-IIA behavior, as NM-IIA-IIB heterodimer formation could complicate results and interpretation (Fig. S1 A). Using super-resolution instant structured illumination microscopy (iSIM), we identified two-puncta structures formed by GFP-tagged NM-IIA, NM-IIAΔNHT, NM-IIA1933x, and NM-IIA1945x. The two bright puncta were presumed to represent head clusters, while the dark central region was presumed to be the bare zone (see zoomed-in view of the white box). Line scans were performed on these two-puncta structures devoid of NM-IIB, as confirmed by NM-IIB immunofluorescence, to assess the impact of NHT truncations on filament size and/or organization. Compared with GFP-NM-IIA, the fluorescence intensity of both the head clusters and bare zone was increased in all truncation variants (Fig. 7 B), suggesting that NHT truncations promote the formation of larger bipolar filaments or filament stacks, consistent with our in vitro observations.

The two-puncta structures identified by iSIM likely contain primarily stacks of two to three parallel filaments rather than single filaments, as previously suggested (Quintanilla et al., 2024; Shutova et al., 2014). To directly visualize the in vivo architecture of individual bipolar filaments formed by NM-IIA NHT variants, we performed immunogold-labeling platinum replica electron microscopy (PREM) in NM-IIA-KO, NM-IIB–knockdown U2OS cells (Fig. S1 B). N-terminally GFP-tagged NM-IIA and its NHT variants were labeled with 18-nm gold particles to mark the NM-IIA heads, while endogenous NM-IIB was labeled with 10-nm gold particles to guide our analysis. Only bipolar filaments formed by GFP-NM-IIA NHT variants that were devoid of NM-IIB were analyzed for their dimensions. We found that WT NM-IIA formed filaments with an average length of 364.8 ± 64.8 nm, whereas filaments formed by NM-IIAΔNHT (382.5 ± 70.0 nm) or NM-IIA1933x (407.3 ± 65.5 nm) were slightly or significantly larger than WT (Fig. 7 C). Consistent with the in vitro data (Fig. 4 F), the bare-zone length of filaments formed by NM-IIAΔNHT (118.8 ± 36.0 nm) and NM-IIA1933x (117.4 ± 36.3 nm) was significantly shorter than that of WT NM-IIA (155.9 ± 39.2 nm), while the width of the filaments—an indicator of overall filament size—for NM-IIAΔNHT (19.4 ± 4.2 nm) and NM-IIA1933x (20.3 ± 4.4 nm) was significantly greater than that for WT NM-IIA (15.3 ± 2.1 nm) (Fig. 7 C).

Together, these iSIM and PREM data indicate that NHT truncations in NM-IIA promote the formation of larger bipolar filaments in vivo, capturing the salient features of the in vitro observations, with inevitable differences in detail that likely reflect the greater complexity of in vivo conditions.

**Figure S2.**
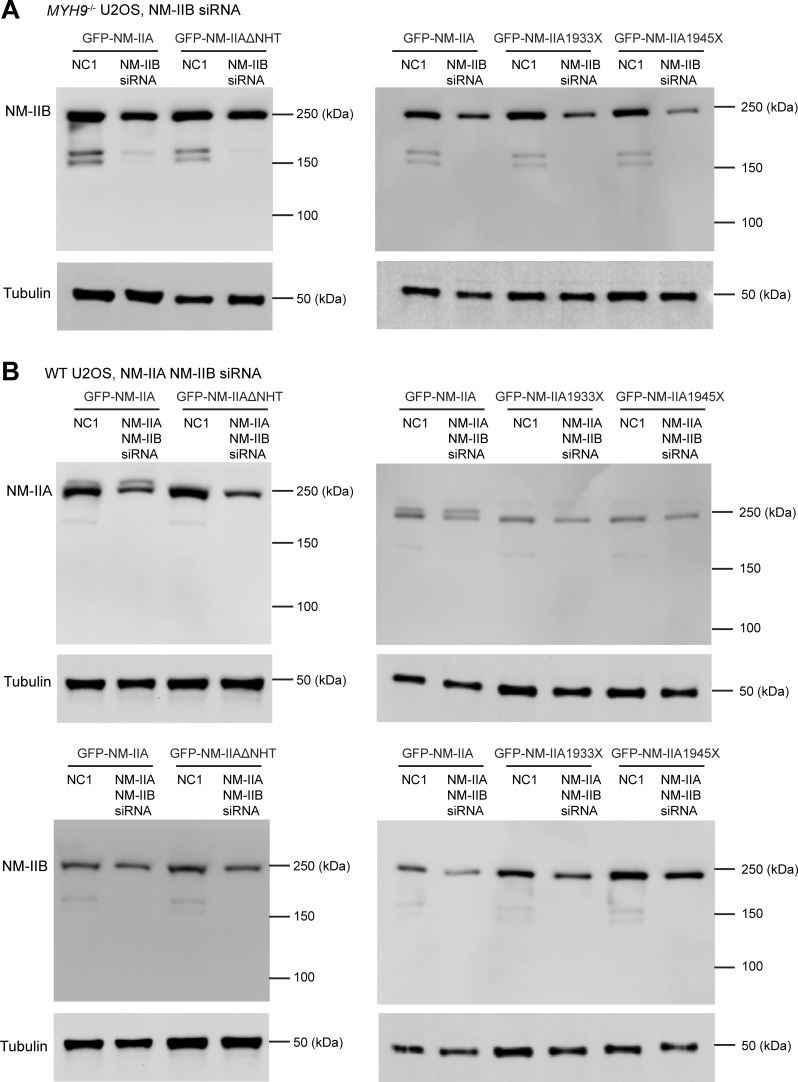
**Western blot analyses of knockdown efficiencies of NM-IIB in NM-IIA-KO U2OS cells and of NM-IIA and NM-IIB in WT U2OS cells expressing GFP-NM-IIA NHT variants used for FRAP analyses. Related to Fig. 7. (A)** Knockdown efficiency of NM-IIB in NM-IIA-KO U2OS cells expressing GFP-NM-IIA NHT variants used for FRAP analyses (related to Fig. 7, D and E). **(B)** Knockdown efficiencies of NM-IIA and NM-IIB in WT U2OS cells expressing GFP-NM-IIA NHT variants used for FRAP analyses (related to Fig. 7, F and G). Source data are available for this figure: SourceData FS2.

To assess whether NHT truncations also affect NM-IIA filament behavior in vivo, we cultured the NM-IIA-KO cells, as described above, expressing different NM-IIA NHT variants with endogenous NM-IIB knocked down (Fig. S2 A). We then photobleached multiple regions in ventral stress fibers and monitored GFP recovery. GFP-NM-IIA recovered rapidly (Fig. 7 D), whereas recovery was minimal for NM-IIAΔNHT and NM-IIA1933x (Fig. 7 D). NM-IIA1945x showed intermediate recovery, slower than WT but greater than NM-IIAΔNHT and NM-IIA1933x (Fig. 7 D). Quantification showed that WT NM-IIA recovered to 36.8 ± 16.0% (*n* = 21) at 290 s after bleach, compared with 21.4 ± 10.0% (*n* = 31) for NM-IIA1945x, 11.7 ± 14.5% (*n* = 29) for NM-IIA1933x, and 5.0 ± 4.0% (*n* = 27) for NM-IIAΔNHT (Fig. 7 E). These data demonstrate that NHT truncations markedly reduce FRAP recovery of NM-IIA within ventral stress fibers in the absence of endogenous NM-IIA, consistent with a defect in filament disassembly.

We next examined the disease-associated stop-codon mutants (1933x and 1945x) in the presence of WT NM-IIA, as these mutations are autosomal dominant. The same GFP-tagged NM-IIA NHT truncation variants were expressed in WT U2OS cells, with endogenous NM-IIA and NM-IIB knocked down to varying degrees (Fig. S2 B). As in NM-IIA-KO cells, but more striking than those, all truncation variants showed markedly reduced recovery than WT NM-IIA, with 1933x and 1945x resembling NHT deletion (Fig. 7 F). WT NM-IIA recovered to 37.2 ± 29.5% (*n* = 31) at 220 s after bleach, whereas 1945x recovered to 14.5 ± 11.2% (*n* = 31), 1933x to 12.8 ± 10.1% (*n* = 32), and NHT deletion to 16.3 ± 12.4% (*n* = 71) (Fig. 7 G). These findings indicate that NHT truncations reduce FRAP recovery of NM-IIA in ventral stress fibers even in the presence of endogenous NM-IIA.

### NHT truncations enhance the ability of NM-IIA to maintain and form filaments when RLC phosphorylation is inhibited

**Figure S3.**
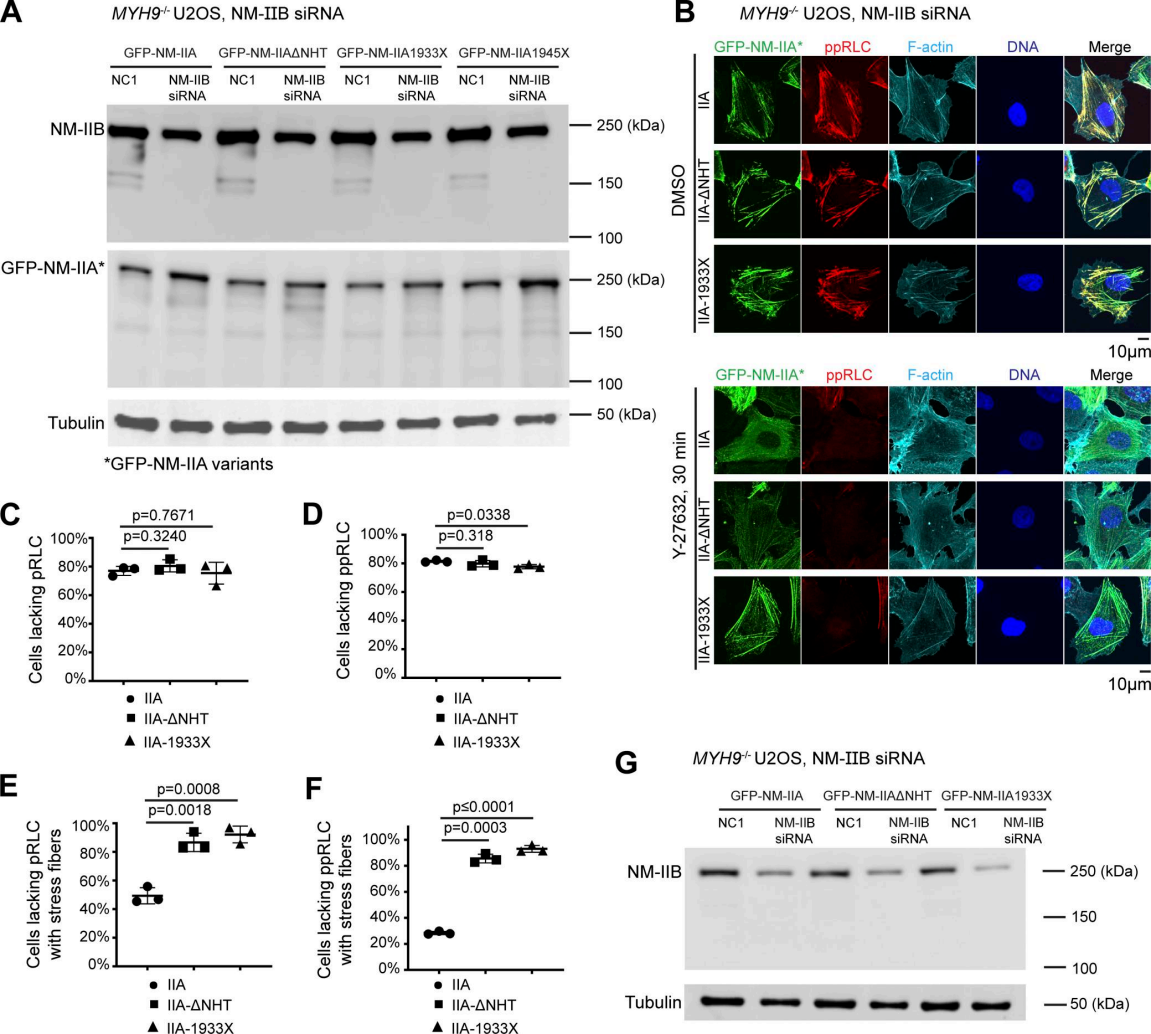
**Western blot and immunofluorescence analyses of NM-IIA-KO cells with NM-IIB knocked down, expressing GFP-NM-IIA NHT variants, and treated with the ROCK inhibitor. Related to Fig. 8. (A)** Knockdown efficiency of NM-IIB and expression levels of GFP-NM-IIA NHT variants (indicated by GFP-NM-IIA*) in NM-IIA-KO U2OS cells used for Y-27632 treatment analyses in live cells (related to Fig. 8 A). **(B–F)** Effects of Y-27632 treatment on RLC phosphorylation (ppRLC) and stress fiber organization in fixed NM-IIB–knockdown, NM-IIA-KO U2OS cells analyzed by light microscopy (related to Fig. 8 D). Representative images of DMSO-treated and Y-27632–treated cells with indicated staining (B) are shown. The percentages of cells lacking pRLC (C) or ppRLC (D), as well as cells lacking pRLC but retaining stress fibers (E) or lacking ppRLC but retaining stress fibers (F), are quantified. **(G)** Knockdown efficiency of NM-IIB in NM-IIA-KO U2OS cells expressing GFP-NM-IIA NHT variants used for Y-27632 treatment analyses by immunogold-labeling PREM (related to Fig. 8, E–G). Source data are available for this figure: SourceData FS3.

**Figure 8.**
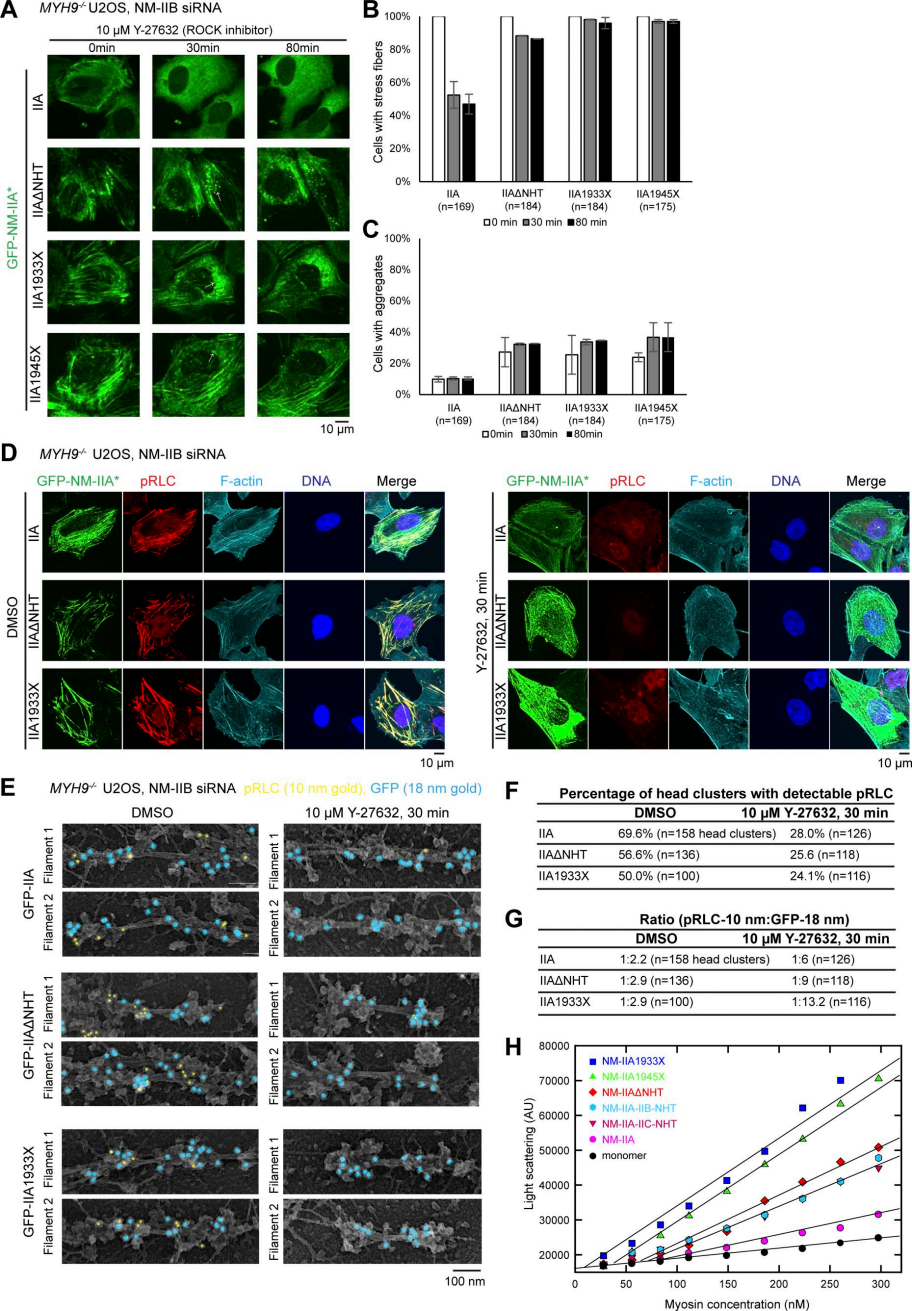
**NHT truncations in NM-IIA slow stress fiber disassembly and enhance assembly when RLC phosphorylation is inhibited. (A)** Stress fiber dynamics in response to inhibition of RLC phosphorylation. 72 h after NM-IIB knockdown by siRNA, NM-IIA-KO U2OS cells expressing GFP-NM-IIA, GFP-NM-IIAΔNHT, GFP-NM-IIA1933X, or GFP-NM-IIA1945X were treated with 10 µM Y-27632 and imaged by time-lapse microscopy at 5-min intervals. Representative images at 0, 30, and 80 min are shown. GFP-NM-IIA* indicates NHT variants. See also Videos 1 and 2. **(B)** Percentage of cells in A retaining stress fibers over time. Data are presented as the mean ± SD from three independent experiments. At time 0, all groups showed 100% stress fiber retention with zero variance; statistical testing was therefore not performed. At 30 min, P values for NM-IIA compared with NM-IIAΔNHT, NM-IIA1933X, and NM-IIA1945X were 0.017, 0.010, and 0.010, respectively. At 80 min, P values for NM-IIA compared with NM-IIAΔNHT, NM-IIA1933X, and NM-IIA1945X were 0.008, 0.001, and 0.004, respectively. **(C)** Percentage of cells in A forming NM-IIA* aggregates over time. Data are presented as the mean ± SD from three independent experiments. At time point 0, P values for comparisons between NM-IIA and NM-IIAΔNHT, NM-IIA1933X, or NM-IIA1945X were 0.081, 0.160, and 0.004, respectively. At 30 min, P values were 9.7 × 10^−6^ for NM-IIA vs. NM-IIAΔNHT, 0.0002 for NM-IIA vs. NM-IIA1933X, and 0.037 for NM-IIA vs. NM-IIA1945X. At 80 min, P values for NM-IIA compared with NM-IIAΔNHT, NM-IIA1933X, and NM-IIA1945X were 0.0006, 0.0004, and 0.037, respectively. **(D)** Effects of Y-27632 treatment on RLC phosphorylation and stress fiber organization. NM-IIB–knockdown, NM-IIA-KO U2OS cells expressing GFP-NM-IIA, GFP-NM-IIAΔNHT, or GFP-NM-IIA1933X were treated with DMSO or 10 µM Y-27632 for 30 min, and stained for pRLC, F-actin, and DNA. GFP is shown in green; pRLC in red; F-actin in cyan; and DNA in blue. **(E)** Immunogold-labeling PREM analysis of NM-II filaments following DMSO or Y-27632 treatment as in D. GFP was labeled with 18-nm gold particles (blue) and pRLC with 10-nm gold particles (yellow). **(F)** Percentage of myosin head clusters containing pRLC from E. **(G)** Ratio of 10-nm gold-labeled pRLC to 18-nm gold-labeled GFP from E. **(H)** CCs for polymerization of NM-IIA NHT variants, determined by light scattering as described in Fig. 6, A and B, except that the assays were performed in the presence of 1 mM ATP.

The presence of larger bipolar filaments and the reduced FRAP recovery of NM-IIA in ventral stress fibers suggest that NM-IIA NHT truncations impair filament disassembly. To test this, we treated NM-IIA-KO, NM-IIB–knockdown U2OS cells (Fig. S3 A) expressing different NM-IIA NHT truncation variants with Y-27632, a ROCK inhibitor known to disassemble NM-II filaments by blocking ROCK-mediated RLC phosphorylation and relieving ROCK-mediated suppression of RLC phosphatase (Davies et al., 2000; Grandy et al., 2022; Narumiya et al., 2000; Yoneda et al., 2005). We then monitored the disassembly of stress fiber–like structures, defined as linear arrays of GFP-NM-IIA variants, by time-lapse microscopy. In cells expressing GFP-NM-IIA, stress fibers disassembled quickly, as indicated by the rapid loss or decrease of stress fiber–associated GFP signal following treatment (Video 1). By 30 min, approximately half of the cells had no discernible stress fibers, while the remainder exhibited only weak structures marked by diminished GFP signal (Fig. 8, A and B). In contrast, stress fiber disassembly was noticeably reduced in cells expressing GFP-tagged NM-IIAΔNHT (Video 2), NM-IIA1933x, or NM-IIA1945x. By 30 min, the majority of the cells expressing any of the NHT truncations contained relatively strong stress fibers (Fig. 8, A and B), and even after 80 min of treatment, most cells expressing the truncation variants continued to display strong stress fibers. Additionally, cells expressing NHT truncations consistently showed a higher level of NM-II aggregation than WT across all treatment time points (Fig. 8 A, arrow; and Fig. 8 C). Thus, NHT truncations lead to reduced stress fiber disassembly and increased NM-IIA aggregation.

**Video 1.** **Time-lapse analysis of GFP-NM-IIA dynamics in NM-IIA-KO U2OS cells in response to Y-27632 treatment (Fig. 8 A).** 72 h after NM-IIB knockdown by siRNA, NM-IIA-KO U2OS cells expressing GFP-NM-IIA were treated with 10 µM Y-27632 immediately after acquisition of the initial time point image and then imaged by time-lapse microscopy at 5-min intervals. Green fluorescence indicates GFP-NM-IIA. This video shows rapid disassembly of GFP-NM-IIA from stress fiber–like structures. Time is displayed as hh:mm, and the movie is shown at 3 frames per second. Related to Fig. 8 A.

**Video 2.** **Time-lapse analysis of GFP-NM-IIAΔNHT dynamics in NM-IIA-KO U2OS cells in response to Y-27632 treatment (Fig. 8 A).** 72 h after NM-IIB knockdown by siRNA, NM-IIA-KO U2OS cells expressing GFP-NM-IIAΔNHT were treated with 10 µM Y-27632 immediately after acquisition of the initial time point image and then imaged by time-lapse microscopy at 5-min intervals. Green fluorescence indicates GFP-NM-IIAΔNHT. This video shows reduced disassembly of GFP-NM-IIAΔNHT from stress fiber–like structures, along with aggregate formation. Time is displayed as hh:mm, and the movie is shown at 3 frames per second. Related to Fig. 8 A.

To determine whether the stress fiber disassembly defects associated with the NHT truncations resulted from insufficient removal of RLC phosphorylation following Y-27632 treatment, we immunostained for mono- and diphosphorylated RLC (pRLC and ppRLC) in fixed NM-IIA-KO, NM-IIB–knockdown U2OS cells (Fig. S1 B). Under DMSO treatment, pRLC and ppRLC were readily detected in cells expressing GFP-NM-IIA, GFP-NM-IIAΔNHT, and GFP-NM-IIA1933X (Fig. 8 D and Fig. S3 B), indicating active RLC phosphorylation. After Y-27632 treatment for 30 min, pRLC staining was absent in 77.0 ± 3.1%, 80.5 ± 4.2%, and 75.5 ± 7.7% of cells expressing GFP-NM-IIA, GFP-NM-IIAΔNHT, and GFP-NM-IIA1933X, respectively (Fig. S3 C), and ppRLC staining was lost in 81.5 ± 0.7% (GFP-NM-IIA), 79.9 ± 2.3% (GFP-NM-IIAΔNHT), and 77.8 ± 1.5% (GFP-NM-IIA1933X) of cells (Fig. S3 D). Thus, treatment with the ROCK inhibitor, as expected, markedly reduced RLC phosphorylation, an effect that is apparently not altered by the NHT truncations.

We then quantified the percentage of pRLC-negative cells that contained stress fibers after Y-27632 treatment. 49.4 ± 5.7% of GFP-NM-IIA–expressing cells had stress fibers, whereas substantially higher proportions were observed in GFP-NM-IIAΔNHT (86.7 ± 6.4%) and GFP-NM-IIA1933X (92.3 ± 5.9%) cells (Fig. S3 E). Similarly, among the ppRLC-negative populations, 28.4 ± 1.2% of GFP-NM-IIA–expressing cells displayed stress fibers, compared with 85.6 ± 3.3% and 93.0 ± 2.6% of GFP-NM-IIAΔNHT and GFP-NM-IIA1933X cells, respectively (Fig. S3 F). These data suggest that NM-IIA can support stress fiber assembly in the absence of RLC phosphorylation, a property that is enhanced by NHT truncations.

The presence of stress fibers in Y-27632–treated cells prompted us to investigate whether NM-IIA and its NHT variants can form or maintain bipolar filaments without RLC phosphorylation at the EM level. Using immunogold labeling of GFP (myosin heads) and pRLC in NM-IIA-KO, NM-IIB–knockdown U2OS cells (Fig. S3 G) for PREM analysis, we found that following Y-27632 treatment, only 28.0% of GFP-NM-IIA, 25.6% of GFP-NM-IIAΔNHT, and 24.1% of GFP-NM-IIA1933X filaments exhibited pRLC labeling, compared with 69.6%, 56.6%, and 50.0%, respectively, in DMSO-treated controls (Fig. 8, E and F). Furthermore, under Y-27632 treatment, the ratio of pRLC to GFP labeling decreased substantially: from 1:2.2 to 1:6 in GFP-NM-IIA, from 1:2.9 to 1:9 in GFP-NM-IIAΔNHT, and from 1:2.9 to 1:13.2 in GFP-NM-IIA1933X (Fig. 8 G). Together, these data indicate that WT NM-IIA can exist as bipolar filaments with little or no detectable pRLC and that this property is enhanced by NHT truncations.

The time-lapse data showed not only rapid disassembly but also de novo assembly of GFP-NM-IIA–containing stress fibers at distinct locations in Y-27632–treated cells (Fig. 8 A; and Videos 1 and 2). This raises the intriguing possibility that unphosphorylated RLC-NM-IIA is able to assemble into filaments in the presence of cellular concentrations of ATP (0.5–5.0 mM) in mammalian cells (Greiner and Glonek, 2021; Huang et al., 2010). To test this in vitro, we polymerized NM-IIA NHT truncation variants at various concentrations in the presence of 1 mM ATP overnight and analyzed them using light scattering. All NHT truncation variants, particularly the disease-associated NM-IIA1933x and NM-IIA1945x, displayed reduced CCs for filament assembly compared with WT (Fig. 8 H). The CCs were 80 nM (NM-IIA WT), 60 nM (NM-IIAΔNHT), 10 nM (NM-IIA1933x), and 35 nM (NM-IIA1945x) (Fig. 8 H).

Given these in vitro CCs and the estimated intracellular concentration of the endogenous NM-IIA HC in U2OS cells (10.8 μM) (Kage et al., 2022), and considering that all GFP-tagged NM-IIA NHT variants were expressed at comparable levels (Fig. S1 A, middle panel), we estimate that GFP-tagged WT NM-IIA was present at ∼18–48% of the endogenous protein level (∼1.9–5.2 μM) (Fig. S2 B, top two panels, lane 1s—“NC1”). At these expression levels, all GFP-tagged NM-IIA variants, particularly the NHT truncations, are expected to assemble de novo filaments, even under conditions where the RLC is dephosphorylated and cellular ATP is present.

Together, these observations suggest that NHT truncations not only impair NM-IIA filament disassembly but also promote de novo NM-IIA assembly in cells when RLC phosphorylation is inhibited.

### NHT truncations promote filament stacking and aggregation in the presence of endogenous NM-IIA

**Figure S4.**
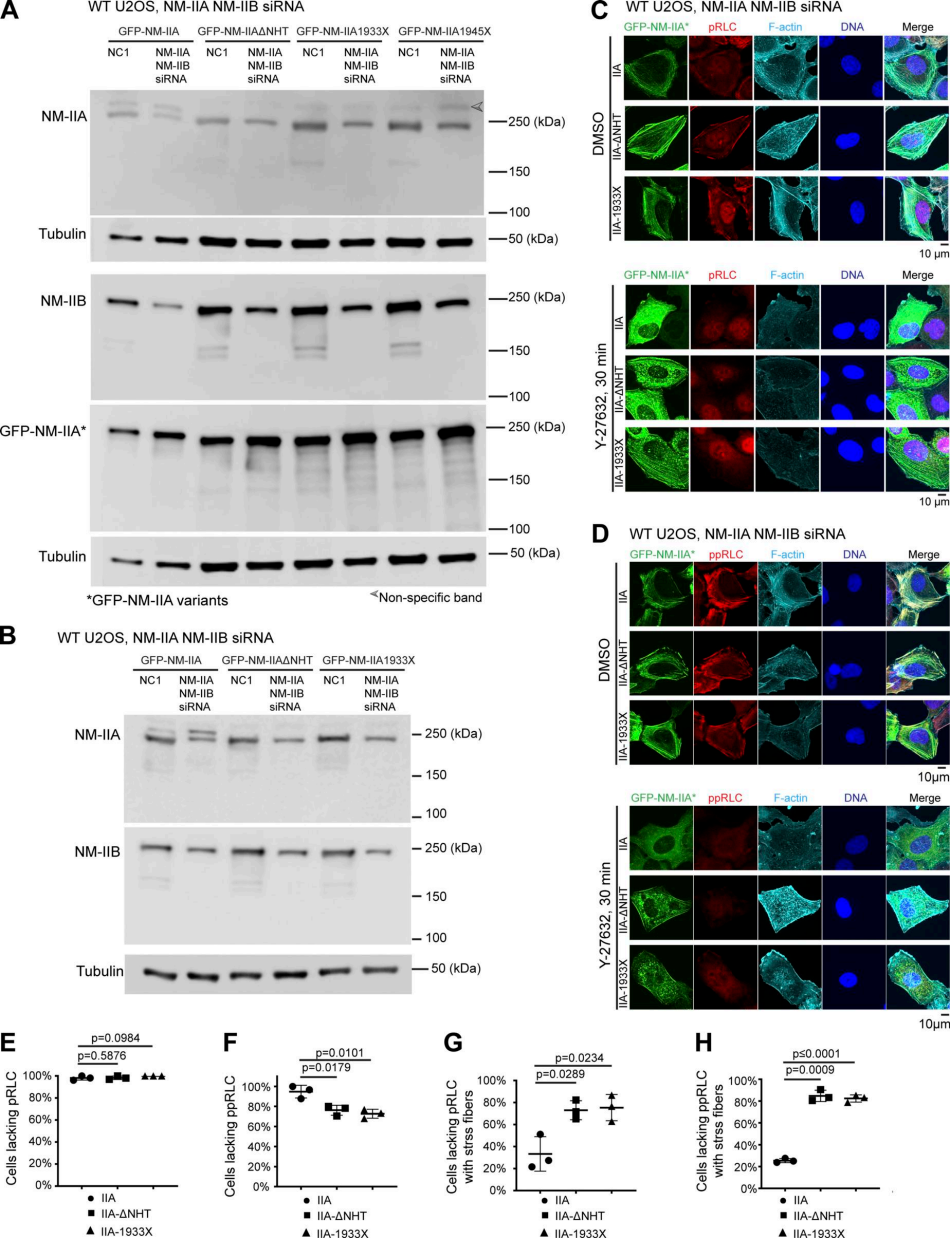
**Western blot and immunofluorescence analyses of WT U2OS cells with NM-IIA/NM-IIB knocked down, expressing GFP-NM-IIA NHT variants, and treated with the ROCK inhibitor. Related to Fig. 9. (A)** Knockdown efficiencies of NM-IIA and NM-IIB and expression levels of GFP-NM-IIA NHT variants (indicated by GFP-NM-IIA*) in WT U2OS cells used for Y-27632 treatment analyses in live cells (related to Fig. 9 A). **(B–H)** Effects of Y-27632 treatment on RLC phosphorylation (pRLC and ppRLC) and stress fiber organization in fixed NM-IIA– and NM-IIB–knockdown WT U2OS cells analyzed by light microscopy. **(B)** Knockdown efficiencies of NM-IIA and NM-IIB in WT U2OS cells used for Y-27632 treatment analyses by immunogold-labeling CL-PREM (related to Fig. 9, D–F), as well as by pRLC and ppRLC immunofluorescence staining (see panels C and D). **(C and D)** Representative images of pRLC (C) or ppRLC (D) staining in DMSO-treated and Y-27632–treated cells are shown. **(E–H)** The percentages of cells lacking pRLC (E) or ppRLC (F), as well as cells lacking pRLC but retaining stress fibers (G) or lacking ppRLC but retaining stress fibers (H), are quantified. Source data are available for this figure: SourceData FS4.

Since disease-associated mutations in *MYH9*, including stop-codon mutations within the NHT of NM-IIA, are autosomal dominant (Asensio-Juarez et al., 2020; Heath et al., 2001; Pecci et al., 2018; Seri et al., 2003), WT and NHT-truncated NM-IIA are expected to co-assemble in patient cells, potentially altering molecular and cellular behaviors. To address this possibility, we examined the phenotypes of WT U2OS cells expressing GFP-tagged NM-IIA NHT variants, which mimic the patient-cell context, to gain further insights into disease mechanisms. Because NM-IIA is highly expressed in U2OS cells, with an approximate NM-IIA:NM-IIB:NM-IIC ratio of 220:14:1 (Kage et al., 2022), we knocked down endogenous NM-IIA to reduce its abundance to levels more comparable to those of GFP-tagged NM-IIA variants (Fig. S4 A). In parallel, we knocked down NM-IIB to minimize complications arising from potential NM-IIA/NM-IIB heterotypic filament formation, though this is unlikely to be a significant issue given the isoform stoichiometry in these cells (Fig. S4 A). Despite variations in the knockdown efficiency of both NM-IIA and NM-IIB, the phenotypes described below remained consistent and specific to the NHT variants.

**Figure 9.**
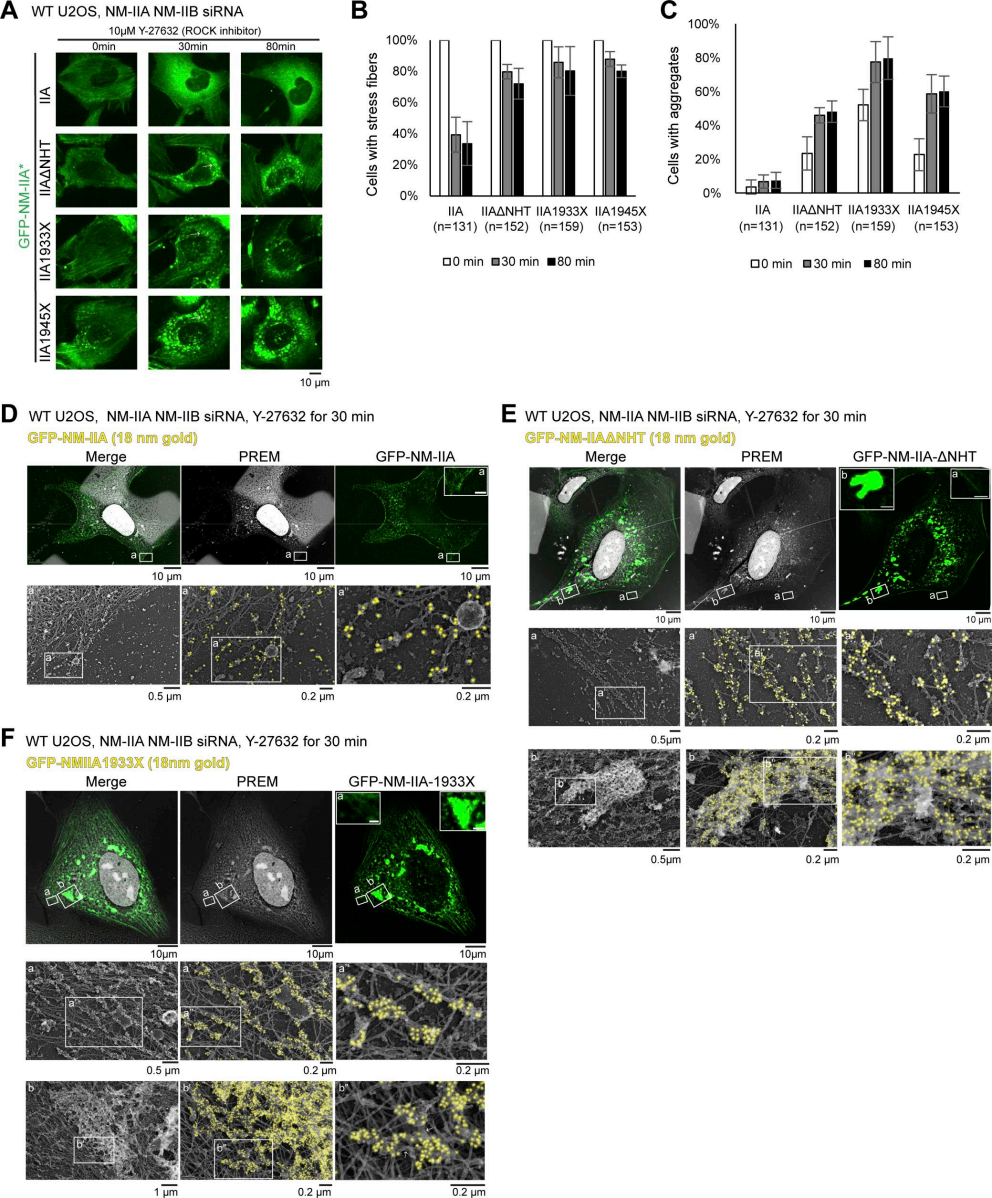
**NHT truncations in NM-IIA slow stress fiber disassembly and enhance myosin filament aggregation. (A)** Stress fiber dynamics in response to inhibition of RLC phosphorylation. 72 h after NM-IIA and NM-IIB knockdown by siRNA, WT U2OS cells expressing GFP-NM-IIA, GFP-NM-IIAΔNHT, GFP-NM-IIA1933X, or GFP-NM-IIA1945X were treated with 10 µM Y-27632 and imaged by time-lapse microscopy at 5-min intervals. Representative images at 0, 30, and 80 min are shown. GFP-NM-IIA* indicates NHT variants. See also Videos 3 and 4. **(B)** Percentage of cells in A retaining stress fibers over time. Data are presented as the mean ± SD from three independent experiments. At time 0, all groups showed 100% stress fiber retention with zero variance; statistical testing was therefore not performed. At 30 min, P values for NM-IIA compared with NM-IIAΔNHT, NM-IIA1933X, and NM-IIA1945X were 0.014, 0.006, and 0.008, respectively. At 80 min, P values for NM-IIA compared with NM-IIAΔNHT, NM-IIA1933X, and NM-IIA1945X were 0.022, 0.019, and 0.023, respectively. **(C)** Percentage of cells in A forming NM-IIA* aggregates over time. Data are presented as the mean ± SD from three independent experiments. At time point 0, P values for comparisons between NM-IIA and NM-IIAΔNHT, NM-IIA1933X, or NM-IIA1945X were 0.056, 0.005, and 0.054, respectively. At 30 min, P values were 0.0004 for NM-IIA vs. NM-IIAΔNHT, 0.006 for NM-IIA vs. NM-IIA1933X, and 0.009 for NM-IIA vs. NM-IIA1945X. At 80 min, P values for NM-IIA compared with NM-IIAΔNHT, NM-IIA1933X, and NM-IIA1945X were 0.001, 0.005, and 0.009, respectively. **(D–F)** Immunogold-labeling CL-PREM analysis of filaments and aggregates formed by GFP-NM-IIA NHT variants in Y-27632–treated WT U2OS cells with NM-IIA and NM-IIB knocked down. Representative images from cells expressing GFP-NM-IIA (D), GFP-NM-IIAΔNHT (E), and GFP-NM-IIA1933X (F) are shown. The GFP fluorescence image is shown on the right, with enlarged boxed regions displayed as inserts at the top right and/or top left corners; the PREM image is shown in the middle, and the merged image on the left. An enlarged view of the boxed region in the PREM panel is shown below. GFP-NM-IIA NHT variants were labeled with 18-nm gold particles (yellow).

Compared with *MYH9*^*−/−*^, NM-IIB–knockdown cells, ROCK inhibition in WT U2OS cells with both NM-IIA and NM-IIB knocked down resulted in more rapid stress fiber disassembly and more pronounced aggregation (Fig. 9 A, arrow) of GFP-tagged NM-IIAΔNHT, NM-IIA1933x, and NM-IIA1945x than of WT NM-IIA. However, stress fibers containing the NHT-truncated NM-IIA variants still disassembled more slowly than those containing WT NM-IIA (Fig. 9, A–C; and Videos 3 and 4). These results suggest that NHT truncations not only slow stress fiber disassembly but also exacerbate NM-IIA mutant aggregation in the presence of endogenous NM-IIA.

**Video 3.** **Time-lapse analysis of GFP-NM-IIA dynamics in WT U2OS cells in response to Y-27632 treatment (Fig. 9 A).** 72 h after siRNA-mediated knockdown of NM-IIA and NM-IIB, WT U2OS cells expressing GFP-NM-IIA were treated with 10 µM Y-27632 immediately after acquisition of the initial time point image and then imaged by time-lapse microscopy at 5-min intervals. Green fluorescence indicates GFP-NM-IIA. This video shows rapid disassembly of GFP-NM-IIA from stress fiber–like structures. Time is shown in hh:mm format, and the movie is shown at 3 frames per second. Related to Fig. 9 A.

**Video 4.** **Time-lapse analysis of GFP-NM-IIAΔNHT dynamics in WT U2OS cells in response to Y-27632 treatment (Fig. 9 A).** 72 h after siRNA-mediated knockdown of NM-IIA and NM-IIB, WT U2OS cells expressing GFP-NM-IIAΔNHT were treated with 10 µM Y-27632 immediately after acquisition of the initial time point image and then imaged by time-lapse microscopy at 5-min intervals. Green fluorescence indicates GFP-NM-IIAΔNHT. This video shows reduced disassembly of GFP-NM-IIAΔNHT from stress fiber–like structures, along with aggregate formation. Time is shown in hh:mm format, and the movie is shown at 3 frames per second. Related to Fig. 9 A.

We then examined the RLC phosphorylation state in WT U2OS cells expressing GFP-tagged NM-IIA NHT variants, with endogenous NM-IIA and NM-IIB knocked down (Fig. S4 B), in the presence or absence of the ROCK inhibitor. In DMSO-treated control cells, pRLC and ppRLC staining was readily detected in cells expressing any of the GFP-tagged NM-IIA NHT variants, indicating active RLC phosphorylation (Fig. S4, C and D). After 30 min of Y-27632 treatment, pRLC signals were efficiently removed in nearly all cells: 98.1 ± 1.9% of GFP-NM-IIA–expressing cells, 98.3 ± 1.6% of GFP-NM-IIAΔNHT–expressing cells, and 100% of GFP-NM-IIA1933X–expressing cells were pRLC-negative based on the same threshold set for the DMSO control (Fig. S4, C and E). Similarly, ppRLC staining was absent in 94.8 ± 6.3%, 76.3 ± 5.0%, and 72.8 ± 4.5% of cells expressing GFP-NM-IIA, GFP-NM-IIAΔNHT, and GFP-NM-IIA1933X, respectively (Fig. S4, D and F). These results suggest that RLC dephosphorylation occurs efficiently even when endogenous NM-IIA and GFP-tagged NM-IIA NHT variants are co-expressed in the same cells.

We also quantified the percentage of pRLC-negative cells that retained stress fibers following Y-27632 treatment and found that 33.3 ± 15.6% of GFP-NM-IIA–expressing cells retained stress fibers, compared with 73.1 ± 8.6% of GFP-NM-IIAΔNHT and 75.3 ± 11.9% of GFP-NM-IIA1933X cells (Fig. S4 G). Similar trends were observed in ppRLC-negative cells, with stress fibers retained in 25.5 ± 1.9% (GFP-NM-IIA), 84.8 ± 5.2% (GFP-NM-IIAΔNHT), and 82.5 ± 3.3% (GFP-NM-IIA1933X) of cells (Fig. S4 H).

Together, these results demonstrate that Y-27632 treatment efficiently dephosphorylates RLC regardless of the presence of endogenous NM-IIA or the expression of NM-IIA NHT truncation variants. The persistent stress fibers in NHT truncation–expressing cells, despite efficient RLC dephosphorylation, suggest that the stress fiber disassembly defects arise from mechanisms independent of RLC phosphorylation.

The aggregates formed in U2OS cells expressing NHT-truncated NM-IIA variants following Y-27632 treatment resembled the inclusion or Dohle-like bodies observed in neutrophils from patients with *MYH9*-RD (Asensio-Juarez et al., 2020; Cai et al., 2024; Pecci et al., 2018; Shen et al., 2024). Whether mutant NM-IIA forms protein or filament aggregates in patient neutrophils, however, remains unclear. To gain insight into the nature of these aggregates, we examined Y-27632–treated U2OS cells using immunogold-labeling correlative light–PREM (CL-PREM). Using GFP fluorescence as a guide, we found that GFP-NM-IIA was partially associated with residual stress fibers and retained a well-defined filamentous structure (Fig. 9 D, a–a”). In cells expressing GFP-NM-IIAΔNHT, a subset of the mutant protein behaved similar to WT NM-IIA, associating with stress fibers and displaying a clear filamentous structure, although often as thickened and stacked filaments compared with the thinner filaments formed by WT NM-IIA (Fig. 9 E, a–a”). Another fraction assembled into large aggregates (Fig. 9 E, b). High-magnification imaging revealed that these aggregates were composed of densely packed NM-IIA filaments (Fig. 9 E, b’ and b”). Similarly, and even more strikingly, in cells expressing GFP-NM-IIA1933X, the mutant protein either associated with stress fibers as thickened and stacked filaments (Fig. 9 F, a–a”) or formed aggregates consisting of bipolar filaments arranged in multiple orientations (Fig. 9 F, b–b”).

Collectively, these data indicate that NHT truncations not only slow NM-IIA filament disassembly but also promote filament aggregation in the presence of endogenous NM-IIA.

### NHT deletion in NM-IIA impairs cell migration

**Figure S5.**
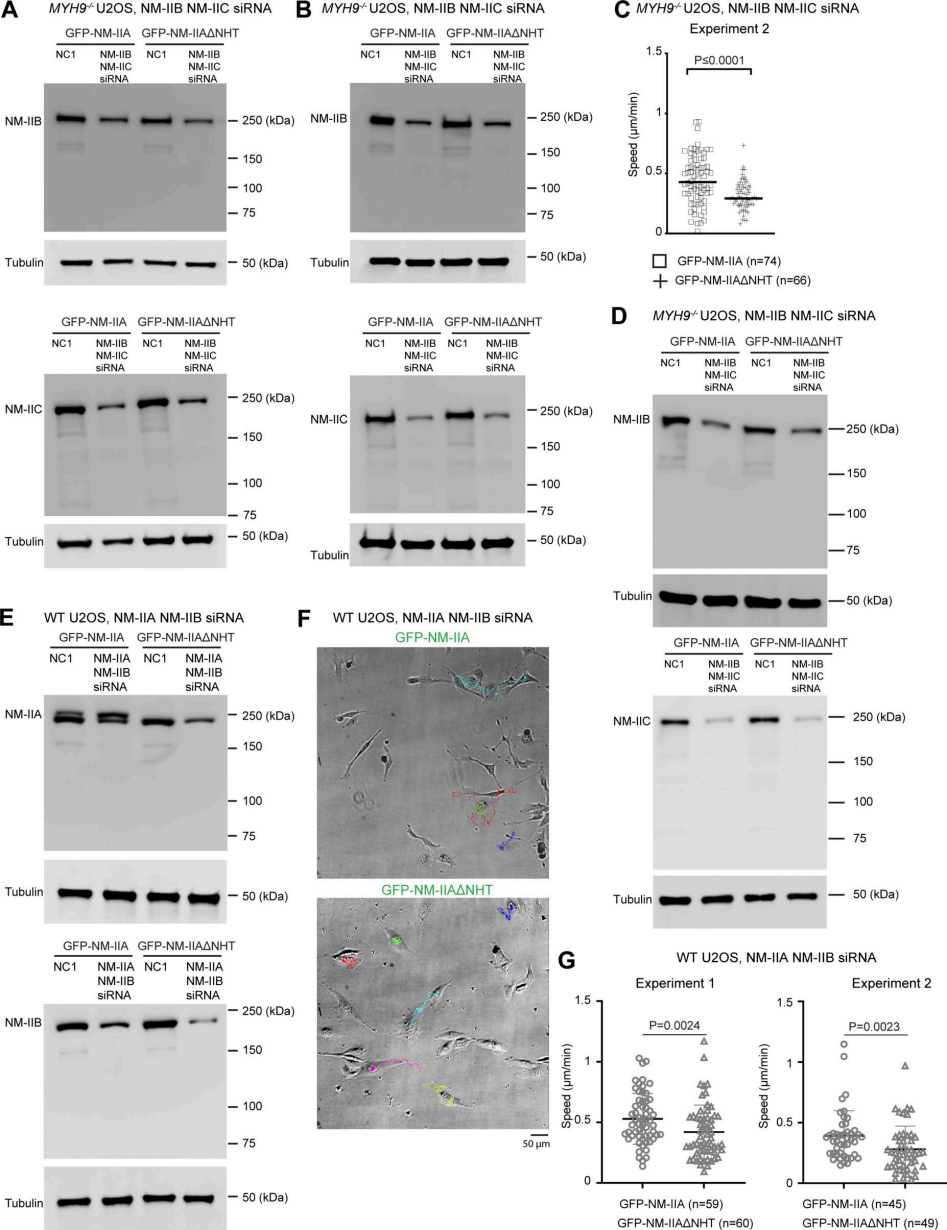
**Western blot and migration analyses of NM-IIA-KO cells with NM-IIB/NM-IIC knocked down and WT cells with NM-IIA/NM-IIB knocked down, expressing GFP-NM-IIA or GFP-NM-IIAΔNHT. Related to Fig. 10. (A and B)** Knockdown efficiencies of NM-IIB and NM-IIC in NM-IIA-KO U2OS cells expressing GFP-NM-IIA or GFP-NM-IIAΔNHT used for experiment 1 (A) (related to Fig. 10, A, B, and D) and experiment 2 (B). **(C)** Quantitative analysis of migration speeds from experiment 2. **(D)** Knockdown efficiencies of NM-IIB and NM-IIC in NM-IIA-KO U2OS cells expressing GFP-NM-IIA or GFP-NM-IIAΔNHT used for analyses of NM-IIA variant and F-actin organization (related to Fig. 10 C). **(E)** Knockdown efficiencies of NM-IIA and NM-IIB in WT U2OS cells expressing GFP-NM-IIA or GFP-NM-IIAΔNHT used for the cell migration analyses shown in F and G. **(F and G)** Migration analyses. Representative imaging fields of the cells from E were analyzed for migration behavior (F). Migration speeds from two independent experiments were quantified and plotted in G. Source data are available for this figure: SourceData FS5.

**Figure 10.**
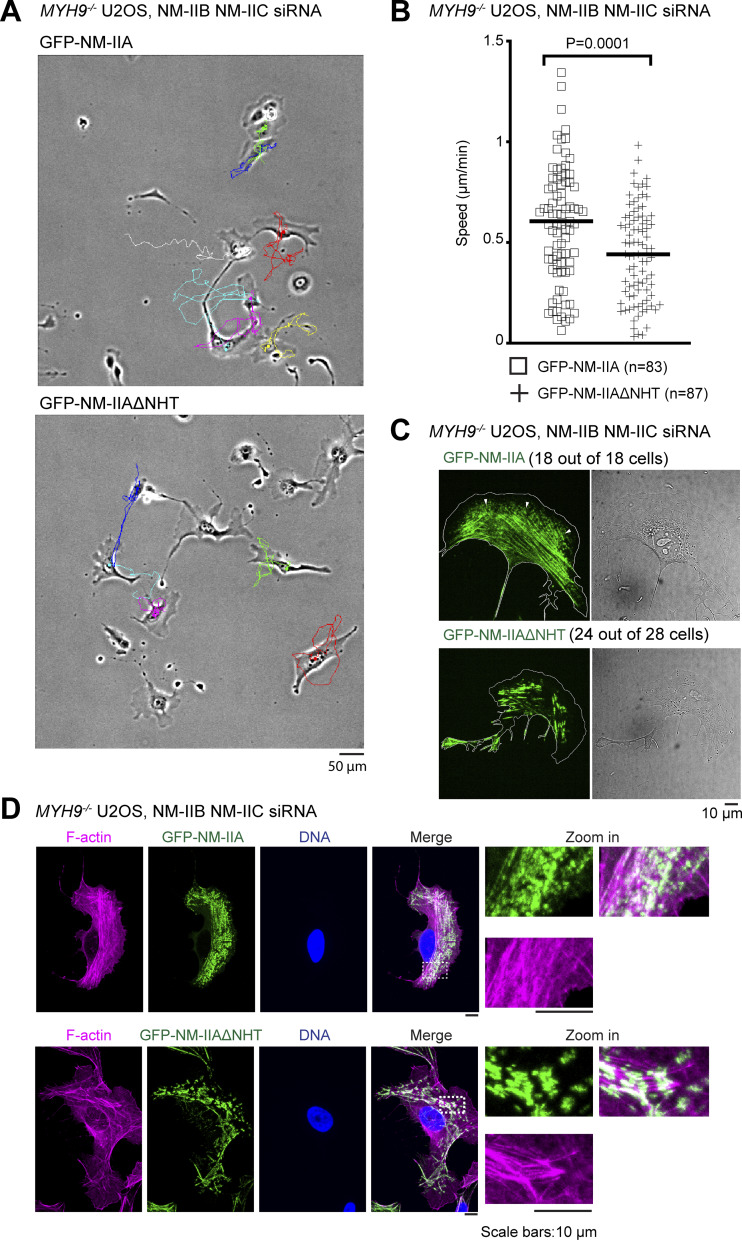
**NHT deletion in NM-IIA impairs cell migration. (A)** NHT deletion reduces migration speed. 72 h after NM-IIB and NM-IIC knockdown by siRNA, NM-IIA-KO U2OS cells expressing GFP-NM-IIA or GFP-NM-IIAΔNHT were seeded onto fibronectin-coated glass-bottom dishes and imaged using a 10× objective to track random cell migration. **(B)** Migration speed of GFP-positive cells from A was measured using the manual tracking function in ImageJ and plotted. Data are presented as the mean ± SD. **(C)** NHT deletion disrupts NM-IIA organization in migrating cells. Cells were treated as in A, except that migrating cells with a clear leading edge were imaged with a 40× objective to visualize NM-IIA organization. **(D)** NHT deletion disrupts both F-actin and NM-IIA organization. NM-IIB– and NM-IIC–knockdown, NM-IIA-KO U2OS cells expressing GFP-NM-IIA or GFP-NM-IIAΔNHT were fixed and stained for F-actin (magenta) and DNA (blue); GFP is shown in green. The boxed region is enlarged and shown on the right.

Cell migration requires stress fiber turnover. As all NM-IIA NHT truncation variants exhibited reduced FRAP recovery, consistent with a filament disassembly defect, we hypothesized that NHT truncation impairs cell migration. To test this, we performed a random cell migration assay. In NM-IIA-KO U2OS cells with knockdowns of NM-IIB and NM-IIC (Fig. S5 A), cells expressing GFP-NM-IIAΔNHT migrated more slowly than those expressing GFP-NM-IIA, with migration speeds of 0.45 ± 0.24 µm/min (*n* = 87) and of 0.60 ± 0.30 µm/min (*n* = 83), respectively (Fig. 10, A and B). This phenotype was reproduced in an independent experiment (Fig. S5, B and C).

To investigate the underlying mechanism, we performed a similar experiment by knocking down both NM-IIB and NM-IIC in NM-IIA-KO U2OS (Fig. S5 D) and then examined the organization of GFP-NM-IIA variants in live migrating cells with a clearly defined leading edge. In all 18 migrating cells expressing GFP-NM-IIA, well-organized myosin filaments were observed, predominantly distributed in front of and along the sides of the nucleus (arrowheads), with a subset of smaller filaments positioned behind the leading edge (Fig. 10 C). In contrast, 24 of 28 migrating cells expressing GFP-NM-IIAΔNHT formed thicker filaments with larger dark zones that were mainly localized at the sides and/or front of the nucleus (Fig. 10 C).

Staining of F-actin and DNA in fixed NM-IIA-KO U2OS cells with knockdowns of NM-IIB and NM-IIC (Fig. S5 A) revealed that nearly all normal- or thicker looking myosin filaments formed by GFP-NM-IIA or GFP-NM-IIAΔNHT were associated with actin filaments in stress fiber–like structures. In contrast, actin filaments at the leading edge contained little or no myosin (Fig. 10 D). These observations indicate that NHT deletion alters the morphology and spatial distribution of stress fibers, likely contributing to the observed migration defect.

Consistent with these findings, similar experiments in WT U2OS with knockdown of endogenous NM-IIA and NM-IIB (Fig. S5 E) showed that GFP-NM-IIAΔNHT–expressing cells also migrated more slowly than those expressing GFP-NM-IIA (Fig. S5, F and G).

Together, these results demonstrate that NHT deletion in NM-IIA impairs cell migration, independent of the presence of the endogenous protein.

## Discussion

This study demonstrates a critical role of the NHT in regulating NM-II filament architecture and function, providing a mechanistic explanation for disease phenotypes caused by stop-codon mutations in the NHT of NM-IIA. More broadly, our analyses suggest that nonhelical terminal regions of structural proteins, including the NM-IIs described here and collagen, may represent a general mechanism for controlling macromolecular assembly and function.

### NHT regulation of NM-IIA filament assembly and architecture in vitro and in vivo

Using full-length NM-IIs containing unphosphorylated RLC for in vitro polymerization assays in the absence of ATP, we found that NHT length is a key determinant of filament size. Specifically, NHT length directly correlates with bare-zone size and inversely correlates with the length and width of mature bipolar filaments. This relationship holds across different NM-II isoforms. The HCs of NM-IIA, NM-IIB, and NM-IIC are 1,960, 1,976, and 2,000 aa long, with NHTs of 33, 43, and 47 residues, respectively. While previous studies on nonstabilized samples found NM-IIA and NM-IIB to be of similar size and NM-IIC to be the smallest (Billington et al., 2013; Liu et al., 2017), our data from 1-ethyl-3-(3-dimethylaminopropyl)carbodiimide hydrochloride (EDC)–stabilized filaments reveal a distinct size hierarchy (IIA > IIB > IIC). A similar pattern is observed among NM-IIA variants in which NHT length or aa composition is altered by swapping NHTs among NM-II isoforms, duplicating the NM-IIA NHT, or replacing the NM-IIA NHT with the nonhelical tail of Myo1C.

We further found that NHT deletion, regardless of the NM-II isoform, resulted in the formation of larger bipolar filaments with reduced bare zones, without affecting the CC or the initial filament assembly process. These filaments tended to stack in a staggered manner, leading to aggregation. Together, these observations support the hypothesis that NHTs protrude from the bare zone and act as a steric block that limits further addition of folded antiparallel tetramers—the principal building blocks of filament assembly, as proposed previously (Liu et al., 2018). In this model, filament size is constrained primarily by the isoform-specific NHT length in concert with the upstream “Assembly Competence Domain” (Breckenridge et al., 2009; Dulyaninova and Bresnick, 2013; Rosenberg et al., 2008; Rosenberg et al., 2013). Removal of the NHT eliminates this steric constraint, allowing additional folded tetramers to laterally associate with the bare zone. This leads to the formation of oversized filaments with reduced bare zones that subsequently stagger into filament aggregates lacking an apparent bare zone.

In vivo, where RLC is presumably subjected to dynamic phosphorylation and dephosphorylation and cellular ATP concentration typically exceeds 1 mM (Greiner and Glonek, 2021; Huang et al., 2010), we observed that all NM-IIA NHT truncation variants formed larger bipolar filaments as revealed by both super-resolution iSIM and immunogold-labeling PREM. These findings mirror the key features observed in vitro. Cells expressing these variants also formed thicker stress fibers with reduced FRAP recovery and increased resistance to ROCK inhibition–induced filament disassembly. Notably, these variants show a propensity to form filament aggregates, particularly during ROCK inhibition–induced filament disassembly. Consistent with these phenotypes, cells expressing NHT-less NM-IIA display compromised migration, underscoring the critical role of the NHT in regulating filament assembly and architecture.

Our findings provide a mechanistic explanation for the pathogenetic phenotypes associated with NM-IIA NHT-linked thrombocytopenia (*MYH*9 1933x and *MYH*9 1945x), which include reduced platelet counts, giant platelets, and mutant NM-IIA–containing inclusion bodies in neutrophils (Asensio-Juarez et al., 2020; Cai et al., 2024; Kunishima et al., 2003; Pecci et al., 2018). Remarkably, mutations in only six codons—two in the motor domain (S96 and R702) and four in the tail domain (R1165, D1424, E1841, and R1933Stop)—account for ∼80% of patient cases (Asensio-Juarez et al., 2020). While other mutations have been examined to varying extents (Cai et al., 2024; Chen et al., 2013; Franke et al., 2005; Hu et al., 2002; Kunishima et al., 2003; Pal et al., 2020; Spinler et al., 2015; Sung et al., 2021; Zhang et al., 2012), the NHT variant (R1933Stop) has not been previously explored. Here, we show that both NM-IIA1933x and NM-IIA1945x, generated by disease-associated stop-codon mutations within the NHT, form enlarged bipolar filaments both in vitro and in vivo. These variants also produce thicker stress fibers with reduced FRAP recovery and filament aggregation, particularly in the presence of the endogenous protein, providing a mechanistic basis for the autosomal dominant nature of these mutations.

NM-IIA likely serves two distinct roles during platelet production (Asensio-Juarez et al., 2020). Megakaryocytes (MKs) differentiate at the endosteum of the bone marrow and subsequently migrate toward the sinusoids, where they release proplatelets. NM-IIA is thought to drive MK migration and the constriction of cellular processes required for proplatelet release into the sinusoids (Asensio-Juarez et al., 2020). Consistent with this model, several *MYH9*-RD missense mutations (R702C, D1424N, and E1841K) impair MK migration (Pal et al., 2020). Among these, the E1841K mutation leads to enlarged bipolar filaments, whereas the others do not (Pal et al., 2020). Given that NHT-less NM-IIA also impairs migration in osteosarcoma-derived U2OS cells (Burmester et al., 2014; Mohseny et al., 2011), it is likely that NM-IIA1933x and NM-IIA1945x compromise platelet production at least in part by impairing MK migration. Moreover, the filament aggregation observed in U2OS cells expressing these variants suggests a reduced pool of functional NM-IIA available to drive proplatelet constriction in MKs, ultimately resulting in giant platelets and reduced platelet count (Asensio-Juarez et al., 2020).

### RLC phosphorylation–dependent control of NM-IIA filament assembly in vivo

The most prevalent and generally accepted model posits that smooth muscle and nonmuscle RLC-unphosphorylated myosin-II monomers adopt a folded 10S conformation with very low actin-activated ATPase activity. Phosphorylation of the RLC at Ser19, often accompanied by Thr18 phosphorylation by kinases such as MLCK and ROCK, promotes a transition to an unfolded 6S conformation with activated ATPase activity and competence for bipolar filament assembly (Amano et al., 1996; Brito and Sousa, 2020; Chinthalapudi and Heissler, 2024; Craig et al., 1983; Smith et al., 1983; Taneja et al., 2021). This model identifies RLC phosphorylation–induced monomer unfolding as a key regulatory step underlying NM-II filament formation.

While the role of RLC phosphorylation in “promoting” filament assembly has been robustly demonstrated in vitro, the conformational state of NM-II monomers is influenced not only by RLC phosphorylation but also by ionic strength. Under physiological ionic strength (150 mM NaCl or KCl), only a fraction of RLC-phosphorylated smooth muscle and NM-II monomers adopt an extended conformation (Craig et al., 1983; Trybus and Lowey, 1984). In addition, RLC-unphosphorylated folded monomers of smooth muscle myosin-II were observed to form antiparallel folded dimers under low-salt conditions (50 mM NaCl) (Trybus and Lowey, 1984) and antiparallel folded tetramers at physiological ionic strength (150 mM KCl) (Trybus and Lowey, 1987). RLC-unphosphorylated NM-II similarly forms antiparallel folded tetramers under comparable conditions, which have been proposed to serve as the principal building blocks of NM-II filaments, leading to the conclusion that RLC phosphorylation is not strictly required for NM-II polymerization in vitro (Liu et al., 2018).

In vivo, RLC phosphorylation is widely assumed to be essential for NM-II filament assembly, largely based on the rapid disassembly of stress fibers in cells treated with MLCK or ROCK inhibitors. Our study shows that when RLC phosphorylation is inhibited by a ROCK inhibitor, stress fibers disassemble rapidly, as expected; however, NM-IIA persists as bipolar filaments, as revealed by immunogold-labeling PREM. These observations indicate that RLC phosphorylation is not essential for NM-II filament assembly or maintenance in vivo. This conclusion is consistent with previous findings that the ratio of pRLC at Ser19 to total RLC is reported to be ∼16% and 31% in interphase and mitotic rat embryonic fibroblast-4A cells, respectively (Yamakita et al., 1994), suggesting that the majority of NM-II bipolar filaments associated with interphase stress fibers are RLC-unphosphorylated.

Supporting this view, GFP-tagged RLC containing nonphosphorylatable mutations at Thr18 and Ser19 was introduced into HeLa cells, suppressing endogenous RLC expression by an unknown mechanism and resulting in 80–95% of total cellular NM-IIA containing the RLC^T18A/S19A^-GFP (Beach et al., 2011). These cells contained abundant stress fibers incorporating the mutant RLC, indicating that unphosphorylated RLC can support bipolar filament assembly. Furthermore, nearly all myosin-II filaments contained nonphosphorylated RLC in relaxed smooth muscle cells (Somlyo et al., 1981). Our study further suggests that deletion of the NHT enhances the ability of RLC-unphosphorylated NM-IIA to assemble into filaments in vivo. This is explained, at least in part, by our in vitro finding that the CC for filament assembly by RLC-unphosphorylated, NHT-less NM-IIA in the presence of 1 mM ATP, conditions that mimic the ROCK inhibitor–treated cellular environment, is lower than that of its WT counterparts.

It is important to emphasize that the ability of RLC-unphosphorylated NM-II to assemble into filaments, when present above its CC in cells, does not conflict with the established conclusion that RLC phosphorylation promotes NM-II filament assembly in the presence of ATP and is essential for activation of the actin-activated ATPase of NM-II. ATP binding and RLC phosphorylation influence NM-II head conformation, thereby modulating the stability of the folded structure. We speculate that ATP binding stabilizes the folded conformation and thus inhibits polymerization. In contrast, RLC phosphorylation loosens the folded structure and promotes polymerization.

In this study, in vitro polymerization was initiated by diluting RLC-unphosphorylated NM-II from high ionic strength buffer (600 mM NaCl) into low ionic strength polymerization buffer (150 mM NaCl) and allowing assembly overnight on ice for EM visualization. Although NM-IIs adopt an extended (6S) conformation in high salt, two scenarios are possible during dilution. Extended monomers could associate intermolecularly to form extended dimers that subsequently polymerize into filaments. Alternatively, extended monomers could undergo intramolecular folding into the compact 10S conformation, which then assembles into filaments, as proposed previously (Liu et al., 2018). Because folding into the 10S structure is intramolecular, whereas dimerization requires intermolecular interactions, folding is typically expected to occur orders of magnitude faster than intermolecular association. Thus, formation of extended dimers during dilution is kinetically disfavored. We therefore propose that upon dilution into low-salt conditions, extended monomers predominantly fold back into the 10S conformation prior to assembly. Consistent with this model, we previously demonstrated that both RLC-phosphorylated and RLC-unphosphorylated NM-IIs assemble into filaments via folded monomers, antiparallel dimers, and tetramers, and that RLC phosphorylation promotes, but is not strictly required for, unfolding and filament assembly in vitro, regardless of the presence of ATP (Liu et al., 2017; Liu et al., 2018).

### NHT sequences as a general mechanism controlling macromolecular assembly

Our study further suggests that the use of the NHT sequences to control macromolecular assembly extends beyond NM-IIs. Similar mechanisms operate in collagen fiber and intermediate filament assembly. Collagen molecules consist of three α-chains that form a triple helix, each with two distinct NHTs at both the N and C termini (Gelman et al., 1979; Holmes et al., 2018; Shoulders and Raines, 2009). The large globular NHTs at both ends prevent procollagen molecules from assembling into fibers within the cell. These NHTs are cleaved by plasma membrane–associated peptidases outside the cell, enabling the formation of tropocollagen triplexes that are competent for fiber assembly. The remaining NHTs, known as telopeptides, regulate subsequent assembly steps, with the C-terminal telopeptide playing a critical role in initiating collagen fiber formation (Gelman et al., 1979; Shayegan et al., 2016).

Similarly, intermediate filament proteins consist of central rod domains flanked by NHTs at both ends. The central rods facilitate lateral tetramer association, whereas the NHTs regulate filament assembly and organization (Bousquet et al., 2001; Etienne-Manneville, 2018; Omary et al., 2006; Ralton et al., 1994; van de Klundert et al., 1993). Together, these examples suggest that NHT-mediated control of NM-II filament assembly represents a general mechanism for regulating macromolecular assembly.

## Materials and methods

### Expression and purification of myosins

The cDNAs of NM-IIA HC (*Homo sapiens* myosin HC 9), NM-IIB HC (*H. sapiens* myosin HC 10 transcript variant 2), and mouse NM-IIC HC (*Mus musculus* myosin HC 14 transcript variant 1) were cloned into pFastBac 1, the Bac-to-Bac plasmid, for expression in Sf9 cells (Invitrogen). All chimeras, deletions, truncations, and point mutations of NM-IIs were constructed using overlap extension PCR (Pogulis et al., 1996). The overlapping length in each extension PCR was 20 bp. Briefly, NheI/KpnI, RsrII/SpeI, and RsrII/KpnI were used to subclone the full-length cDNAs of NM-IIA, NM-IIB, and NM-IIC, respectively, into pFastBac 1, resulting in pFastBacNM-IIA, pFastBacNM-IIB, and pFastBacNM-IIC. RsrII/KpnI was used for subcloning all overlap extension PCR products carrying substitutions (NHTs of NM-IIB and NM-IIC, AM1C tail sequence, and the extended NM-IIA NHT sequence), and truncations (1933x and 1945x) into pFastBacNM-IIA. MluI/SpeI was used to subclone the overlap extension PCR products carrying the NM-IIC NHT and NHT deletion sequences into pFastBac2B. AatII/KpnI was used for subcloning the overlap extension PCR products carrying the NM-IIB NHT and NHT deletion sequences into pFastBac2C. A FLAG tag (DYKDDDDK) was added to the N termini of the HCs to facilitate the purification of the recombinant myosins. All mutations were confirmed by DNA sequencing. The cDNAs of NM-II RLC (*H. sapiens* NM-II, sequence ID NP_291024.1) and ELC (*M. musculus* NM-II, sequence ID NP_034990.1) were cloned into pFastBac 1. The baculoviruses for expressing the HCs, RLC, and ELC were constructed according to the manufacturer’s product manual (Invitrogen). Recombinant full-length myosins were produced by co-expression of the HC and two light-chain baculoviruses in Sf9 cells. The recombinant NM-IIs were purified using anti-FLAG resin (Sigma-Aldrich) affinity chromatography, as described previously (Billington et al., 2013; Liu et al., 2017). After dialysis against 10 mM MOPS (pH 7.0), 600 mM NaCl, and 1 mM dithiothreitol (DTT) to remove the FLAG peptides, the purified myosins were aliquoted and stored in liquid nitrogen.

### Protein concentration assay and electrophoresis

Protein concentrations were determined using the Bradford reagent (Bio-Rad), with purified myosin as the standard. The protein concentration of myosin was determined by UV absorbance using the formula: myosin (mg/ml) = A280/0.56. SDS-PAGE was performed according to standard procedures on NuPAGE gels (Invitrogen).

### Light-scattering assay of myosin assembly

Myosin samples in 600 mM NaCl were cleared by centrifugation at 300,000 × *g* for 15 min at 4°C using a Beckman TL-100 centrifuge. Myosins were polymerized overnight on ice in a solution containing 150 mM NaCl, 10 mM MOPS (pH 7.0), 2 mM MgCl_2_, 0.1 mM EGTA, and 1 mM DTT, with or without 1 mM ATP. The samples were then warmed to room temperature for 30 min, and light scattering was measured at 20°C using a photon technology international (PTI) fluorimeter. Excitation and detection were performed at 365 nm, with a slit width of 0.5 nm.

### Polymerization

Myosin samples in 600 mM NaCl were cleared by centrifugation at 300,000 × *g* for 15 min at 4°C using a Beckman TL-100 centrifuge. Myosins (300 nM) were polymerized either overnight on ice or for 4 s at room temperature in a solution containing 10 mM MOPS (pH 7.0), 150 mM NaCl, 2 mM MgCl_2_, 1 mM DTT, and 0.1 mM EGTA, in the absence of ATP. The sole exception was for reactions containing varying concentrations of NM-IIA NHT variants, in which 1 mM ATP was included during overnight polymerization to determine CCs for filament assembly (Fig. 8 H). For negative-staining EM, 4-s polymerized myosins were fixed with 0.1 mM glutaraldehyde for 1 min and overnight polymerized myosins were stabilized with 0.1 mM EDC (#22980; Thermo Fisher Scientific) for 30 min at room temperature.

### Sedimentation assay of myosin assembly

Quantification of polymerized myosin by sedimentation in Fig. 6 C was performed as described previously (Liu et al., 2017).

### Negative-staining EM

Samples from the overnight or 4-s polymerization reactions described above were diluted to a myosin concentration of ∼200 nM. Aliquots (4 μl) were applied to UV light–pretreated, carbon-coated copper grids and stained with 1% uranyl acetate. Micrographs were acquired at room temperature using a JEOL 1200EX II microscope. Filament lengths and widths were measured with MetaMorph software (MetaMorph, Inc.).

### Cell lines and culture conditions

A U2OS cell line was a gift from Dr. Matthew Good (University of Pennsylvania, Philadelphia, PA, USA), and NM-IIA-KO cell line was a gift from Dr. Henry Higgs (Dartmouth College, Hanover, NH, USA). U2OS cells were maintained in DMEM supplemented with 10% FBS and 1xPlasmocin prophylactic (preventative antibiotic for *Mycoplasma* infection) at 37°C in the presence of 5% CO_2_.

### Lentivirus packaging and transduction of U2OS cells

Lentivirus was packaged by cotransfecting HEK293T cells with the plasmids pLJM1-GFP-NM-IIA, pLJM1-GFP-NM-IIAΔNHT, pLJM1-GFP-NM-IIA1933X, or pLJM1-GFP-NM-IIA1945X, along with the packaging plasmids pMDLg/pRRE, pRSV-Rev, and the VSV-G envelope–expressing vector pMD2.G, using Lipofectamine 3000 transfection reagent according to the manufacturer’s instructions (Thermo Fisher Scientific). The medium was changed to DMEM with 10% FBS 8 h after transfection. The supernatant containing lentivirus was collected at 32 and 56 h after transfection. The supernatants from these two time points were combined and filtered through a 0.45-μm syringe filter. The filtered lentivirus-containing medium was concentrated 13X using Lenti-X Concentrator (cat#631232; Takara). The concentrated lentivirus solution was then added to U2OS cells in DMEM containing 10% FBS, with polybrene added to a final concentration of 8 μg/ml. The expression of tagged proteins was checked 48 h after transduction.

### siRNA-mediated knockdown of gene expression in U2OS cells

siRNA-mediated knockdown of gene expression was performed following the reverse transfection protocol provided by the Lipofectamine RNAiMAX Transfection Reagent manufacturer, with some modifications. Briefly, RNAi duplex–Lipofectamine RNAiMAX complexes were prepared by combining 150 μl of Opti-MEM with 6 μl of Lipofectamine RNAiMAX and mixing it with 150 μl of Opti-MEM containing 60 pmol of siRNA. The combined solution was incubated at 23°C for 20 min and then mixed with 3 ml of U2OS cells at a concentration of 0.05 × 10^6^ cells/ml. After 72 h, cells were either used for time-lapse live-cell imaging or fixed for staining.

### Immunostaining of U2OS cells

U2OS cells were seeded onto 15-mm fibronectin-coated coverslips and incubated in DMEM containing 10% BSA in a 12-well plate. The cells were fixed with 4% paraformaldehyde for 10 min and then washed three times with PBS. Following fixation, the cells were permeabilized in PBS containing 0.2% Triton X-100 for 10 min, after which they were washed three times with PBS. The coverslips were then blocked in PBS containing 1% BSA for 30 min. Primary rabbit anti-NM-IIB (3404S; Cell Signaling), anti-pRLC (pS19) (3671; Cell Signaling), or anti-ppRLC (pT18S19) (3674; Cell Signaling) was diluted in PBS containing 1% BSA and applied to the coverslips, which were incubated overnight at 4°C. Afterward, the cells were washed three times with PBS and incubated with AF568-conjugated donkey anti-rabbit antibody (A10042; Thermo Fisher Scientific), diluted 1:500 with AF647-conjugated phalloidin (A22287; Invitrogen; at 1:40 dilution) in PBS containing 1% BSA, at 23°C for 2 h. Finally, the cells were washed three times with PBS and mounted in medium containing DAPI (H-1200; Vector Laboratories).

### F-actin staining

For Fig. 10 D, U2OS cells were seeded onto 15-mm fibronectin-coated coverslips and incubated in DMEM containing 10% BSA in a 12-well plate. The cells were fixed with 4% paraformaldehyde for 10 min and then washed three times with PBS. Following fixation, the cells were permeabilized in PBS containing 0.2% Triton X-100 for 10 min, after which they were washed three times with PBS. The coverslips were then blocked in PBS containing 1% BSA for 30 min. Afterward, the cells were incubated with AF568-conjugated phalloidin (A12380; Invitrogen; at 1:40 dilution) in PBS containing 1% BSA, at 23°C for 1 h. Finally, the cells were washed three times with PBS and mounted in mounting medium containing DAPI (H-1200; Vector Laboratories).

### Western blot

Western blotting was performed as described previously (Wang et al., 2023). The primary antibodies used were rabbit anti-NM-IIA (3403S; Cell Signaling), rabbit anti-NM-IIB (3404S; Cell Signaling), rabbit anti-NM-IIC (8189S; Cell Signaling), and rabbit anti-GFP (50430-2-AP; Proteintech), each diluted at 1:1,000. The mouse anti-α-tubulin antibody (DM1a; Sigma-Aldrich) was diluted at 1:3,000.

### Live-cell imaging and data analysis

Time-lapse imaging analysis was performed as described previously, with slight modifications (Wang et al., 2023). Images were acquired using a Nikon microscope (Eclipse Ti2-U) with either a Nikon 10×/NA objective or a Nikon 40× oil objective, and a Yokogawa spinning-disk confocal scanner unit (model CSU-X1). A Photometrics EMCCD camera (Evolve 512 Delta) was used for image capture. Solid-state lasers were used for excitation (488 nm for GFP, 561 nm for RFP, and 405 nm for DAPI). The imaging system was controlled by MetaMorph version 7.10.4.431 (Molecular Devices) or VisiView software (by Visitron). U2OS cells were grown in a fibronectin-coated, glass-bottom chambered dish with DMEM supplemented with 10% FBS and were imaged at 37°C in an Okolab stage-top incubation chamber with 5% CO_2_. For the Y-27632 treatment experiment, the medium was replaced with fresh medium containing 10 µM Y-27632 after the first time point was captured. A sum or single projection was created using NIH ImageJ (1.53t). Data analyses were performed using Microsoft Excel, GraphPad Prism 9.4.1, and R (ver. 3.0.1).

### Random cell migration assay

U2OS cells were seeded onto a fibronectin-coated, glass-bottom chambered dish with DMEM supplemented with 10% FBS and were imaged with a 10× lens at 37°C in an Okolab chamber with 5% CO_2_. The nuclear position of each GFP-positive cell was tracked using the manual tracking function in NIH ImageJ (1.53t). Data analyses were performed using Microsoft Excel and GraphPad Prism 9.4.1.

### FRAP

Cell culture and sample preparation for FRAP analysis were the same as described above in “Live-cell imaging and data analysis.” The imaging system used consisted of a spinning-disk confocal scanner unit (model CSU-X1, Yokogawa) and a microscope (model IX83, Olympus) equipped with a 100×/1.40 oil Olympus objective and pco.edge 4.2 bi sCMOS cameras. VisiView software (Visitron) was used for hardware control and image acquisition. Diode lasers (488 nm for GFP and 561 nm for RFP) were used for excitation. For photobleaching, a 405-nm laser was applied to a defined subcellular region. A single focal plane was created and analyzed with NIH ImageJ. In ImageJ, a polygon was drawn encircling the bleached area to calculate the integrated density within the area over time. Data were analyzed with Microsoft Excel.

### VT-iSIM super-resolution imaging

Fixed and immunofluorescently stained U2OS cells were imaged using the VisiTech (VT)-iSIM super-resolution imaging system (VisiTech International, Inc.). Images were acquired with an Olympus microscope (model IX71 inverted microscope, Olympus) equipped with an Olympus UAPON 100x TIRF/NA 1.49 oil immersion objective (Olympus) and a VT-iSIM confocal scan head (VisiTech International, Inc.). The Hamamatsu ORCA-Quest qCMOS camera (model C15550-20UP, Hamamatsu Photonics) was used for image capture. The imaging system was controlled by MetaMorph (Molecular Devices). Images were taken with 21 z-stacks, each 0.2 μm thick. The Microvolution deconvolution plugin in ImageJ was used to deconvolve the VT-iSIM images. GFP signal distribution was calculated using the line scan function in ImageJ. Data analyses were performed using Microsoft Excel and R (ver. 3.0.1).

### Immunogold-labeling PREM

Cells were extracted with 0.5% Triton X-100 in PEM buffer (100 mM PIPES-KOH, pH 6.9, 1 mM MgCl_2_, 1 mM EGTA) supplemented with 10 µM Taxol. After extraction, samples were rinsed three times in PEM buffer containing 1 µM Taxol. Next, cells were incubated in G buffer (50 mM MES-KOH [pH 6.3], 0.1 mM CaCl_2_, 2 mM MgCl_2_, 0.5 mM DTT) containing 0.4 μg/ml of gelsolin (gift from A. Weber and T. Svitkina, University of Pennsylvania, Philadelphia, PA, USA) for 20 min, followed by two washes in PEM buffer. Extracted cells were then fixed in 2% glutaraldehyde in 0.1 M cacodylate buffer for 20 min. Fixation was quenched using 2 mg/ml NaBH_4_ in PBS. Cells were incubated with goat anti-GFP (ab5450; Abcam) and rabbit anti-NM-IIB (3404S; Cell Signaling) primary antibodies for 2 h at room temperature, washed three times with PBS, and then incubated overnight at 23°C with 18-nm gold-conjugated donkey anti-goat (705-215-147; Jackson ImmunoResearch) and 10-nm gold-conjugated donkey anti-rabbit (ab39597; Abcam) secondary antibodies. After three washes with immunogold buffer (20 mM Tris-HCl, pH 8.0, 0.5 M NaCl, and 0.05% Tween-20) with 0.1% BSA, cells were postfixed in 2% glutaraldehyde and processed for PREM as previously described (Shutova et al., 2012). Samples were imaged using a JEM-1011 transmission electron microscope (JEOL) operated at 100 kV. Images were acquired with an ORIUS 832.10W CCD camera (Gatan) and are shown with inverted colors (black–white inversion), with or without pseudocoloring. Color labeling and image overlays were performed in Adobe Photoshop (Adobe Systems), as previously described (Shutova et al., 2012).

For GFP and pRLC double staining, cells were extracted with 0.5% Triton X-100 in PEM buffer (100 mM PIPES-KOH, pH 6.9, 1 mM MgCl_2_, 1 mM EGTA) supplemented with 10 µM Taxol and PhosSTOP (4906837001; Roche). After extraction, samples were rinsed three times with PEM buffer containing 1 µM Taxol and PhosSTOP. Cells were then incubated for 30 min in G buffer (50 mM MES-KOH, pH 6.3, 0.1 mM CaCl_2_, 2 mM MgCl_2_, 0.5 mM DTT) containing 0.4 µg/ml gelsolin (a gift from A. Weber and T. Svitkina, University of Pennsylvania, Philadelphia, PA, USA) and rabbit anti-pRLC primary antibody (3671; Cell Signaling) and PhosSTOP. Samples were washed twice with PEM buffer with Taxol and PhosSTOP and subsequently fixed with 2% glutaraldehyde in 0.1 M cacodylate buffer for 20 min. Fixation was quenched with 2 mg/ml NaBH_4_ in PBS. Cells were then incubated with rabbit anti-pRLC primary antibody (3671; Cell Signaling) overnight at 4°C, followed by incubation with goat anti-GFP primary antibody (ab5450; Abcam) for 2 h at room temperature. After three washes with PBS, cells were incubated for 10 h at 23°C with 10-nm gold-conjugated donkey anti-rabbit secondary antibody (ab39597; Abcam). Subsequently, cells were incubated overnight at 23°C with 18-nm gold-conjugated donkey anti-goat antibody (705-215-147; Cell Signaling) together with 10-nm gold-conjugated donkey anti-rabbit antibody (ab39597; Abcam). After three washes with immunogold buffer, cells were postfixed in 2% glutaraldehyde and processed for PREM as previously described (Shutova et al., 2012).

### Immunogold-labeling CL-PREM

CL-PREM was performed as described previously (Yang and Svitkina, 2019). Briefly, U2OS cells expressing fluorescent protein(s) were grown on homemade coverslips with fiducial marks that were coated with fibronectin. After extraction, samples were rinsed three times in PEM buffer containing 1 µM Taxol. Next, cells were incubated in G buffer containing gelsolin for 20 min, followed by two washes in PEM buffer cells, and then were fixed with 0.2% glutaraldehyde, quenched with 2 mg/ml NaBH_4_ in PBS, and washed with PBS. For fluorescence microscopy, cells in glass-bottomed dishes were imaged by spinning-disk confocal microscopy with a ×40 objective. After imaging, cells were incubated with goat anti-GFP primary antibody (ab5450; Abcam) and rabbit anti-NM-IIB primary antibody (3404S; Cell Signaling) for 1.5 h at 23°C. Following three washes with PBS, cells were incubated overnight at 23°C with 18-nm gold-conjugated donkey anti-goat (705-215-147; Jackson ImmunoResearch) and 10-nm gold-conjugated donkey anti-rabbit (ab39597; Abcam) secondary antibodies. After three washes with immunogold buffer, the samples were postfixed with 2% glutaraldehyde and processed for PREM.

PREM samples were examined using JEM 1011 transmission EM (JEOL) operated at 100 kV. Images were acquired by an ORIUS 832.10 W CCD camera (Gatan) and presented in inverted contrast. Correlative light microscopy and PREM images were aligned using Adobe Photoshop by rotating and proportionally enlarging fluorescence images until the best match of individual cell regions was achieved. As a result of sample shrinkage after critical point drying, perfect alignment of whole cells could not be achieved, but images of smaller cell regions could be reliably matched.

### Statistical analysis

For statistical analysis, each dataset was first tested for normality using the Shapiro–Wilk test. If the data followed a normal distribution, a two-tailed t test was performed (Figs. 8, B and C; Fig. 9, B and C; Fig. 10 B; Fig. S3, C–F; Fig. S4, F–H; and Fig. S5 C); if not, a two-sided Mann–Whitney U test was applied (Fig. 7 C, Fig. S4 E, and Fig. S5 G).

### Online supplemental material

Fig. S1 shows western blot analyses of NM-IIB knockdown efficiency and GFP-tagged NM-IIA NHT variant expression levels in NM-IIA-KO U2OS cells. Fig. S2 shows western blot analyses of knockdown efficiencies of NM-IIB in NM-IIA-KO U2OS cells and of NM-IIA and NM-IIB in WT U2OS cells expressing GFP-NM-IIA NHT variants used for FRAP analyses. Fig. S3 shows western blot and immunofluorescence analyses of NM-IIB–knockdown, NM-IIA-KO cells expressing GFP-NM-IIA NHT variants treated with the ROCK inhibitor. Fig. S4 shows western blot and immunofluorescence analyses of NM-IIA– and NM-IIB–knockdown WT U2OS cells expressing GFP-NM-IIA NHT variants treated with the ROCK inhibitor. Fig. S5 shows western blot and migration analyses of NM-IIB– and NM-IIC–knockdown, NM-IIA-KO cells and NM-IIA– and NM-IIB–knockdown WT cells expressing GFP-NM-IIA or GFP-NM-IIAΔNHT. Videos 1 and 2 show stress fiber dynamics of NM-IIA-KO U2OS cells expressing GFP-NM-IIA or GFP-NM-IIAΔNHT, respectively, in response to Y-27632 treatment. Videos 3 and 4 show stress fiber dynamics of WT U2OS cells expressing GFP-NM-IIA or GFP-NM-IIAΔNHT, respectively, in response to Y-27632 treatment. Y-27632 was added between time points 1 and 2 in all videos.

## Supplementary Material

SourceData F1 is the source file for Fig. 1.

SourceData FS1 is the source file for Fig. S1.

SourceData FS2 is the source file for Fig. S2.

SourceData FS3 is the source file for Fig. S3.

SourceData FS4 is the source file for Fig. S4.

SourceData FS5 is the source file for Fig. S5.

## Data Availability

The data supporting the findings of this study are included in the paper and its supplemental information and will be available upon request. This paper does not contain any original code.
