# Supplementary material for: Role of the nonhelical tailpiece of myosin-II in regulating filament architecture and function
Source: J Cell Biol. 2026 Jun 25;225(8):e202501234. doi: 10.1083/jcb.202501234 (PMC13296757; doi:10.1083/jcb.202501234)

Figure 1A

Red rectangles indicate lanes taht were cropped, with brightness and contrast adjusted, and then used for Figure 1A.

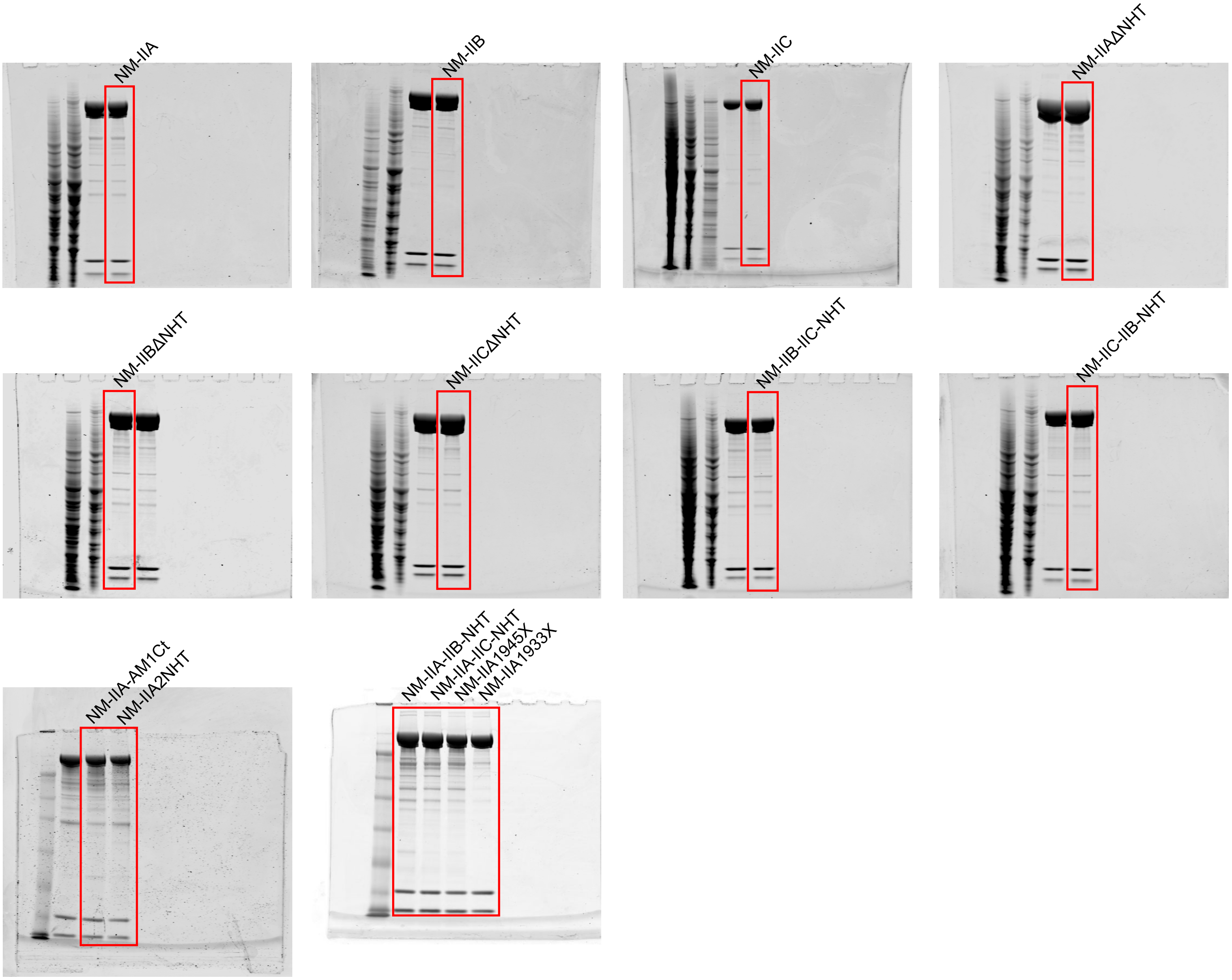

Supplement: SourceData F1 — is the source file for Fig. 1. [file jcb_202501234_sourcedataf1.pdf]
