# Supplementary material for: Role of the nonhelical tailpiece of myosin-II in regulating filament architecture and function
Source: J Cell Biol. 2026 Jun 25;225(8):e202501234. doi: 10.1083/jcb.202501234 (PMC13296757; doi:10.1083/jcb.202501234)
Supplement: SourceData FS2 — is the source file for Fig. S2. [file jcb_202501234_sourcedatafs2.pdf]

Figure S2A

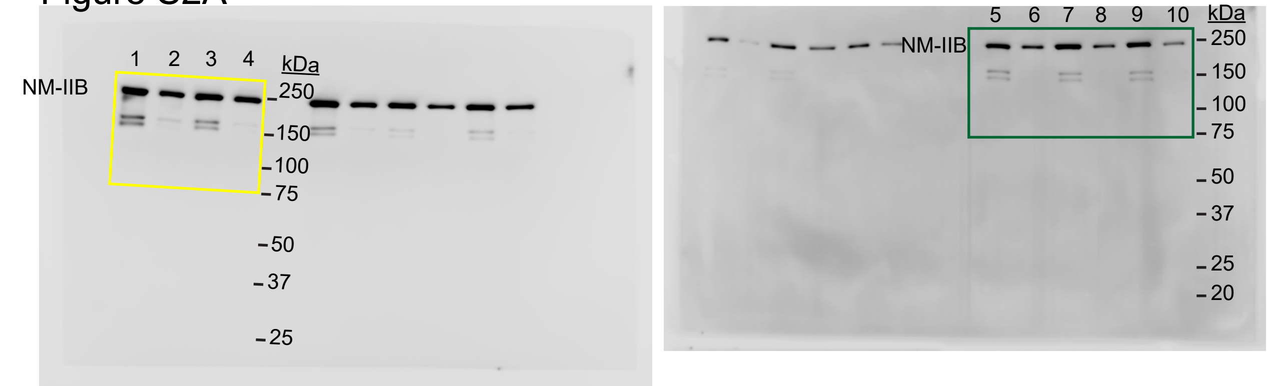

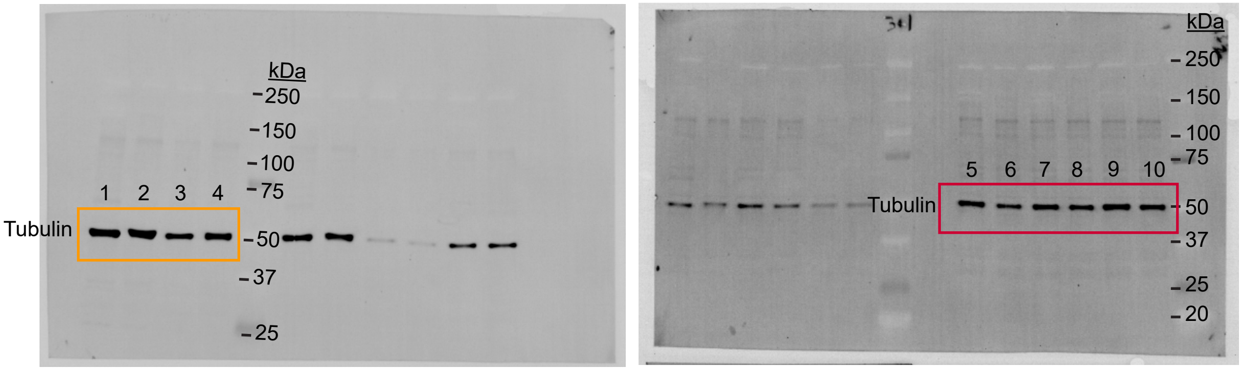

MYH9$^{-/-}$ U2OS, NM-IIB siRNA
1: GFP-NM-IIA NC1
2: GFP-NM-IIA NM-IIB siRNA
3: GFP-NM-IIAΔNHT NC1
4: GFP-NM-IIAΔNHT NM-IIB siRNA
5: GFP-NM-IIA NC1
6: GFP-NM-IIA NM-IIB siRNA
7: GFP-NM-IIA1933X NC1
8: GFP-NM-IIA1933X NM-IIB siRNA
9: GFP-NM-IIA1945X NC1
10: GFP-NM-IIA1945X NM-IIB siRNA

Yellow box indicates cropped region used in Figure S2A (left NM-IIA panel)
Orange box indicates cropped region used in Figure S2A (left Tubulin panel)
Green box indicates cropped region used in Figure S2A (right NM-IIB panel)
Red box indicates cropped region used in Figure S2A (right Tubulin panel)

Figure S2B

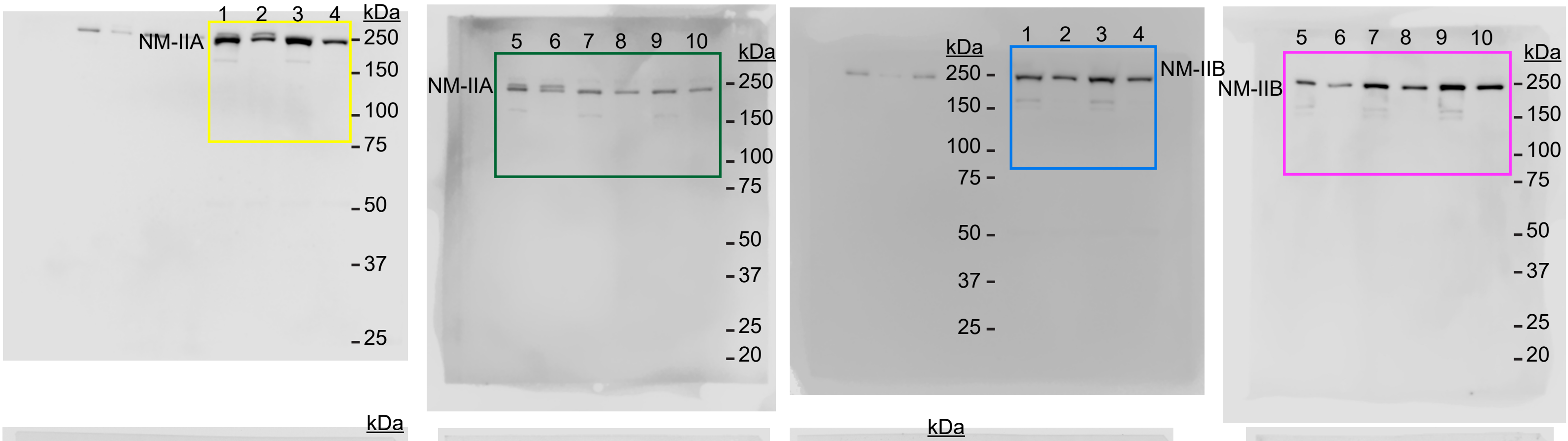

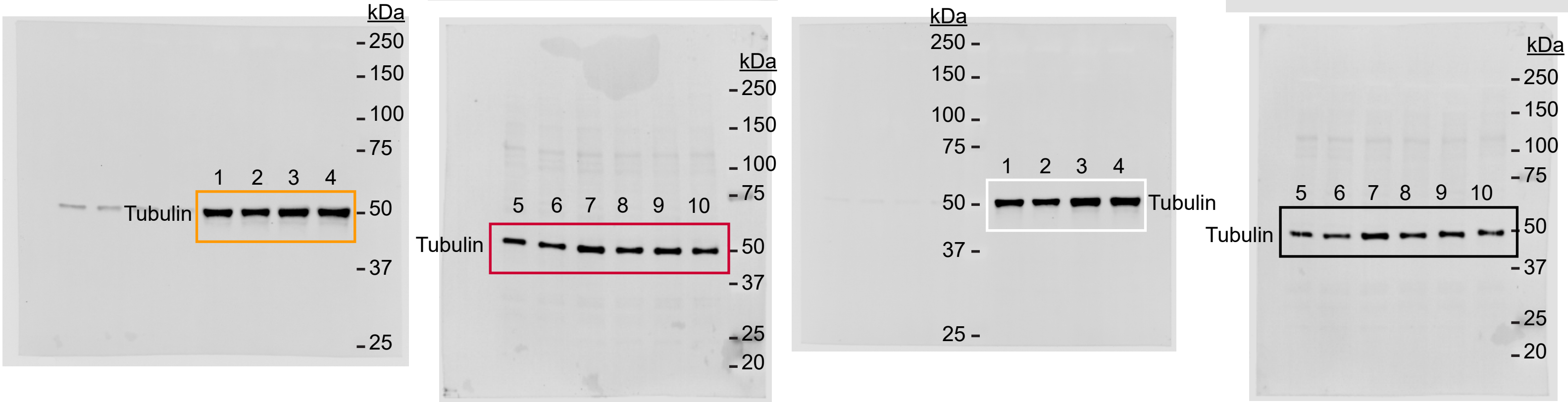

Yellow box indicates cropped region used in Figure S2B (left top NM-IIA panel)
Orange box indicates cropped region used in Figure S2B (Left top Tubulin panel)
Green box indicates cropped region used in Figure S2B (right top NM-IIB panel)
Red box indicates cropped region used in Figure S2B (right top Tubulin panel)
Blue box indicates cropped region used in Figure S2B (left bottom NM-IIA panel)
White box indicates cropped region used in Figure S2B (Left bottom top Tubulin panel)
Magenta box indicates cropped region used in Figure S2B (right bottom NM-IIB panel)
Black box indicates cropped region used in Figure S2B (right top Tubulin panel)

WT U2OS, NM-IIA NM-IIB siRNA
1: GFP-NM-IIA NC1
2: GFP-NM-IIA NM-IIA NM-IIB siRNA
3: GFP-NM-IIAΔNHT NC1
4: GFP-NM-IIAΔNHT NM-IIA NM-IIB siRNA
5: GFP-NM-IIA NC1
6: GFP-NM-IIA NM-IIA NM-IIB siRNA
7: GFP-NM-IIA1933X NC1
8: GFP-NM-IIA1933X NM-IIA NM-IIB siRNA
9: GFP-NM-IIA1945X NC1
10: GFP-NM-IIA1945X NM-IIA NM-IIB siRNA
