# Supplementary material for: Role of the nonhelical tailpiece of myosin-II in regulating filament architecture and function
Source: J Cell Biol. 2026 Jun 25;225(8):e202501234. doi: 10.1083/jcb.202501234 (PMC13296757; doi:10.1083/jcb.202501234)
Supplement: SourceData FS3 — is the source file for Fig. S3. [file jcb_202501234_sourcedatafs3.pdf]

Figure S3A

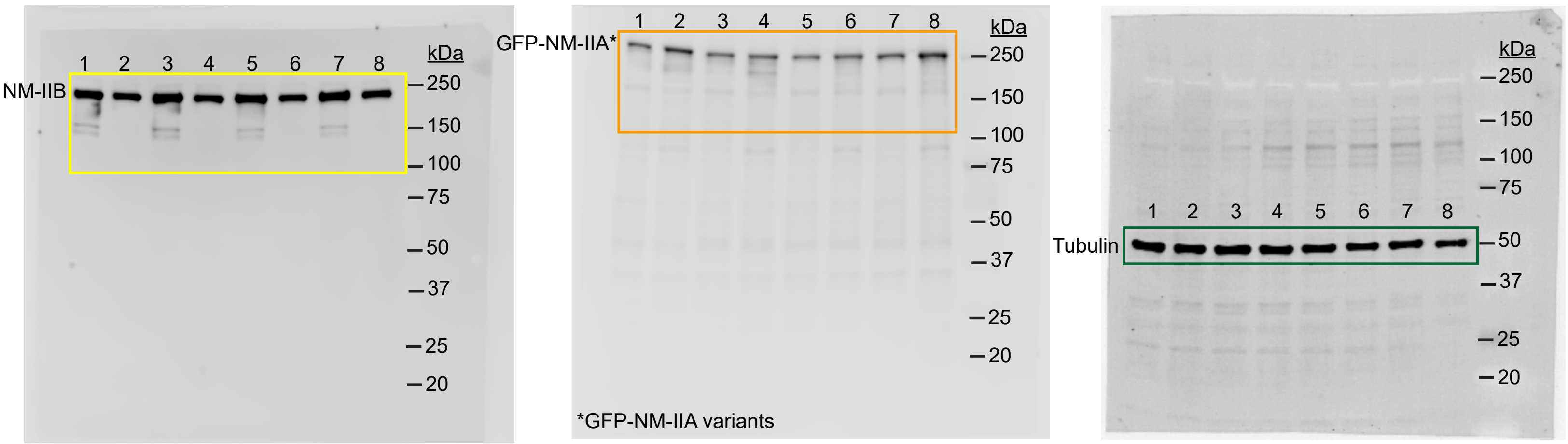

Yellow box indicates cropped region used in Figure S3A (top NM-IIB panel)
Orange box indicates cropped region used in Figure S3A (middle GFP-NM-IIA panel)
Green box indicates cropped region used in Figure S3A (bottom Tubulin panel)

MYH9$^{-/-}$ U2OS, NM-IIB siRNA
1: GFP-NM-IIA NC1
2: GFP-NM-IIA NM-IIB siRNA
3: GFP-NM-IIAΔNHT NC1
4: GFP-NM-IIAΔNHT NM-IIB siRNA
5: GFP-NM-IIA1933X NC1
6: GFP-NM-IIA1933X NM-IIB siRNA
7: GFP-NM-IIA1945X NC1
8: GFP-NM-IIA1945X NM-IIB siRNA

Figure S3G

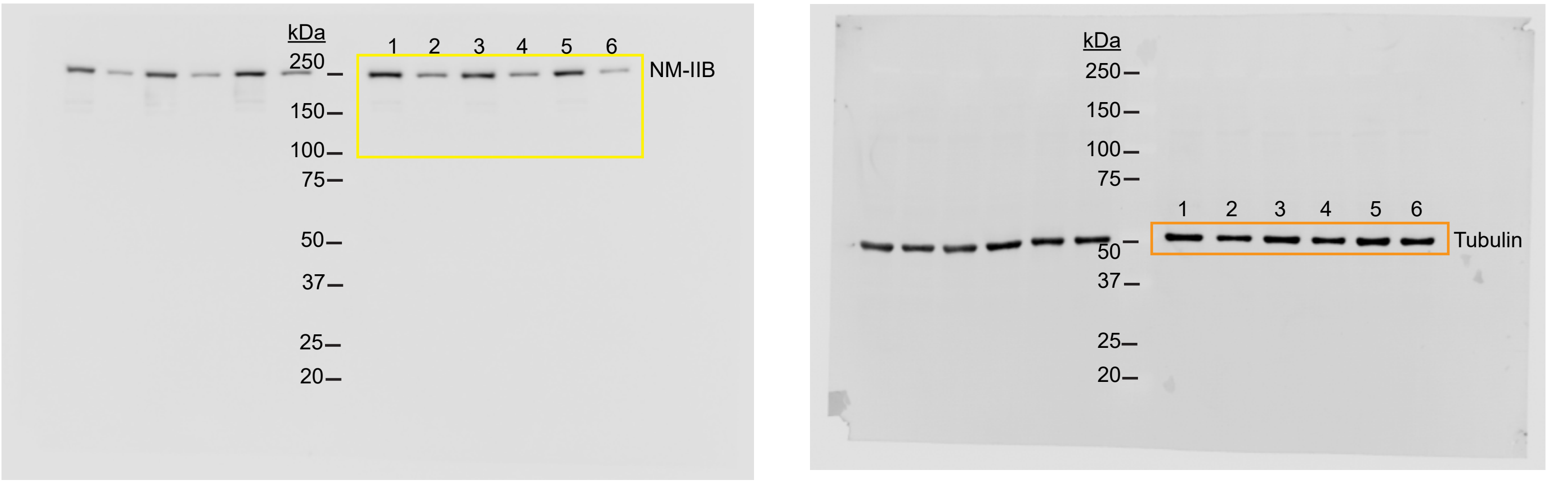

Yellow box indicates cropped region used in Figure S3G (top NM-IIB panel)
Orange box indicates cropped region used in Figure S3G (bottom Tubulin panel)

MYH9$^{-/-}$ U2OS, NM-IIB siRNA
1: GFP-NM-IIA NC1
2: GFP-NM-IIA NM-IIB siRNA
3: GFP-NM-IIAΔNHT NC1
4: GFP-NM-IIAΔNHT NM-IIB siRNA
5: GFP-NM-IIA1933X NC1
6: GFP-NM-IIA1933X NM-IIB siRNA
