# Supplementary material for: Role of the nonhelical tailpiece of myosin-II in regulating filament architecture and function
Source: J Cell Biol. 2026 Jun 25;225(8):e202501234. doi: 10.1083/jcb.202501234 (PMC13296757; doi:10.1083/jcb.202501234)
Supplement: SourceData FS5 — is the source file for Fig. S5. [file jcb_202501234_sourcedatafs5.pdf]

Figure S5A and B

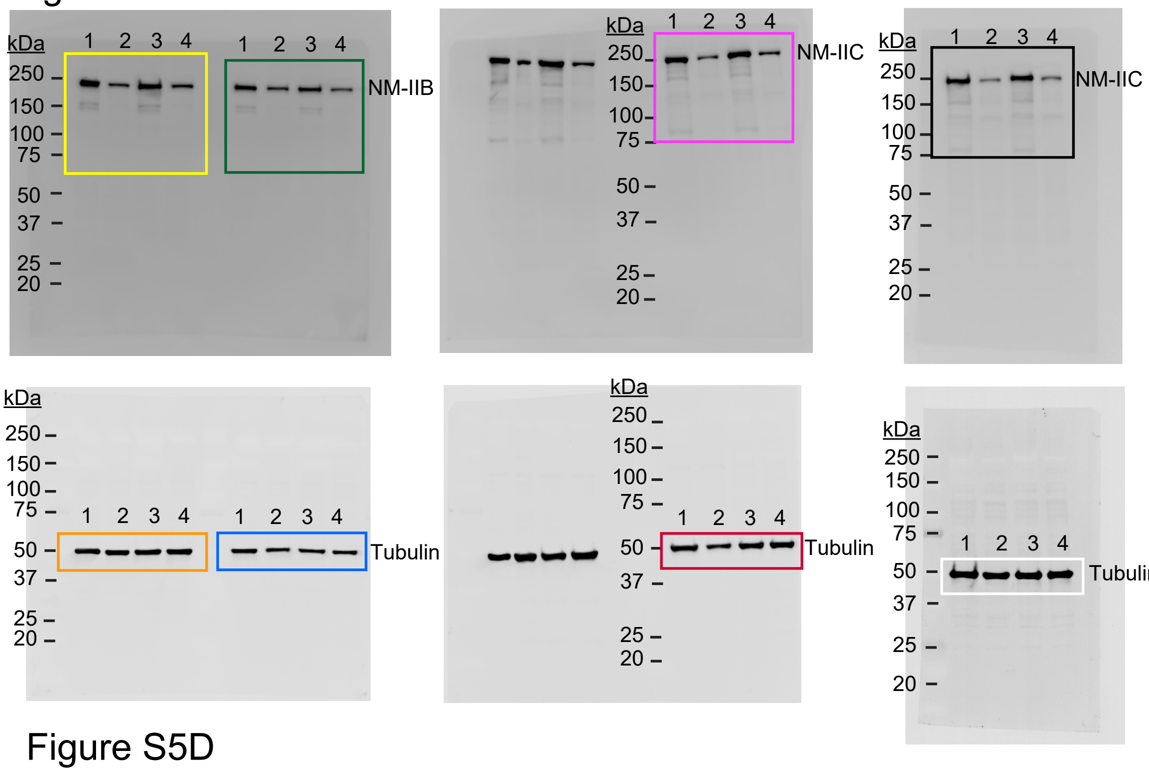

Yellow box indicates cropped region used in Figure S5B (top NM-IIB panel)
Orange box indicates cropped region used in Figure S5B (middle Tubulin panel)
Green box indicates cropped region used in Figure S5A (top NM-IIB panel)
Blue box indicates cropped region used in Figure S5A (middle Tubulin panel)
Magenta box indicates cropped region used in Figure S5A (middle NM-IIC panel)
Red box indicates cropped region used in Figure S5A (bottom Tubulin panel)
Black box indicates cropped region used in Figure S5B (middle NM-IIC panel)
White box indicates cropped region used in Figure S5B (bottom Tubulin panel)

MYH9$^{-/-}$ U2OS, NM-IIB NM-IIC siRNA
1: GFP-NM-IIA NC1
2: GFP-NM-IIA NM-IIB NM-IIC siRNA
3: GFP-NM-IIAΔNHT NC1
4: GFP-NM-IIAΔNHT NM-IIB NM-IIC siRNA

Figure S5D

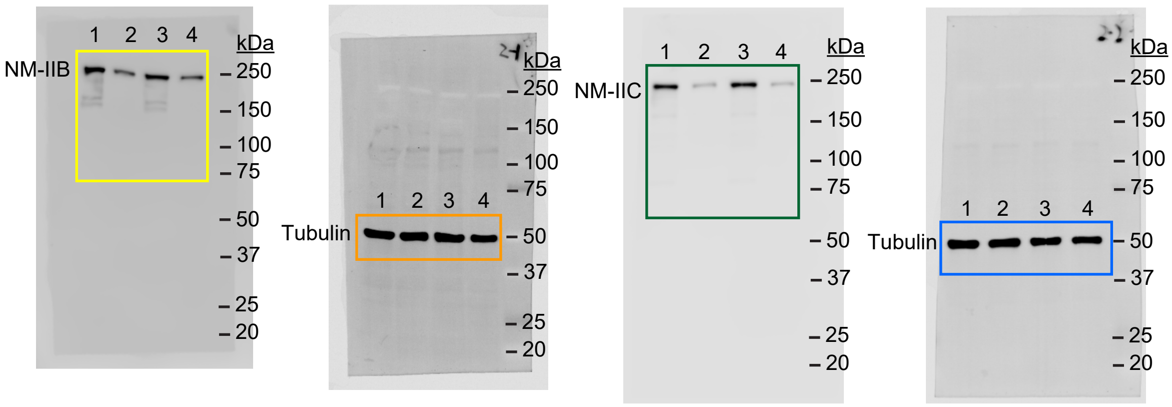

Yellow box indicates cropped region used in Figure S5D (top NM-IIB panel)
Orange box indicates cropped region used in Figure S5D (middle Tubulin panel)
Green box indicates cropped region used in Figure S5D (middle NM-IIC panel)
Blue box indicates cropped region used in Figure S5D (bottom Tubulin panel)

MYH9$^{-/-}$ U2OS, NM-IIB NM-IIC siRNA
1: GFP-NM-IIA NC1
2: GFP-NM-IIA NM-IIB NM-IIC siRNA
3: GFP-NM-IIAΔNHT NC1
4: GFP-NM-IIAΔNHT NM-IIB NM-IIC siRNA

Figure S5E

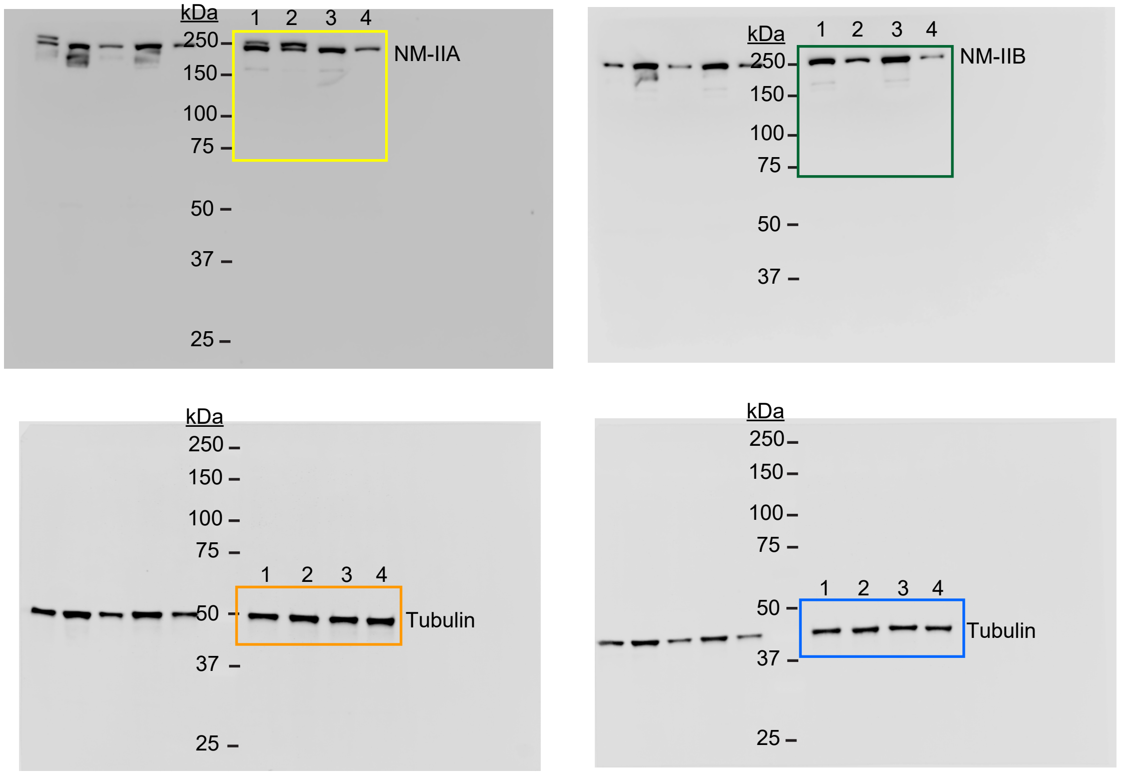

Yellow box indicates cropped region used in Figure S5E (top NM-IIA panel)
Orange box indicates cropped region used in Figure S5E (middle Tubulin panel)
Green box indicates cropped region used in Figure S5E (middle NM-IIB panel)
Blue box indicates cropped region used in Figure S5E (bottom Tubulin panel)

MYH9$^{-/-}$ U2OS, NM-IIA NM-IIB siRNA
1: GFP-NM-IIA NC1
2: GFP-NM-IIA NM-IIA NM-IIB siRNA
3: GFP-NM-IIAΔNHT NC1
4: GFP-NM-IIAΔNHT NM-IIA NM-IIB siRNA
